# Ser/Thr phosphorylation of *Mycobacterium tuberculosis* type II RelK toxin by PknK destabilizes TA interaction and interferes with toxin neutralization

**DOI:** 10.1128/mbio.01068-25

**Published:** 2025-06-17

**Authors:** Shafinaz Rahman Sarah, Abhishek Garg, Sadiyah Afroz, Shaleen Korch, Arjun Ray, Amita Gupta, Vandana Malhotra

**Affiliations:** 1 Department of Biochemistry, Sri Venkateswara College, University of Delhi 208742 , New Delhi, India; 2 Department of Biochemistry, University of Delhi South Campus 93081, New Delhi, India; 3 Department of Computational Biology, Indraprastha Institute of Information Technology 243095, Delhi, India; 4 Department of Pharmacology, Midwestern University 3541, Glendale, Arizona, USA; Institut Pasteur, Paris, France; Sri Guru Gobind Singh Tricentenary University, Gurugram, Haryana, India

**Keywords:** *Mycobacterium tuberculosis*, toxin-antitoxin modules, persistence, post-translational modification, Ser/Thr phosphorylation, PknK, regulation

## Abstract

Toxin-antitoxin (TA) modules represent genetic elements implicated in bacterial persistence. *Mycobacterium tuberculosis* encodes 90+ TA modules, the majority of which are type II, comprising of a toxin component and an antitoxin counterpart that neutralizes the toxin. Under stressful environments, the antitoxin is degraded, releasing the toxin which then acts to halt cellular growth. Towards elucidating the underlying regulatory mechanisms that govern a synchronized TA cellular program, we explored the regulation of type II TA modules by post-translational modification. *In silico* analysis revealed that ~85% of *M. tuberculosis* TA proteins possess potential Ser/Thr phosphorylation sites, implicating them as targets for mycobacterial Ser/Thr protein kinases (STPKs). We demonstrate that members of the RelBE family interact with PknK, a stress-responsive STPK using the mycobacterial protein fragment complementation (M-PFC) assay and are subjected to Ser/Thr phosphorylation *in vitro*. LC-MS/MS confirmed multiple sites of phosphorylation in the RelJK module. Results from molecular dynamics simulations, *in vitro* binding, and co-expression studies with RelJK proteins indicate that the secondary structure changes associated with Thr77 phosphorylation in RelK toxin compromise its binding to the RelJ antitoxin. Substitution of Thr77 with alanine or glutamate in RelK toxin resulted in poor binding to the RelJ antitoxin, allowing a partial rescue of cells co-expressing wild-type RelJ antitoxin and RelK phosphorylation-deficient (T77A) or phosphomimetic (T77E) mutant toxins vs wild-type RelJK proteins. These findings implicate the RelK Thr77 residue at the toxin-antitoxin interaction interface and, more importantly, establish toxin phosphorylation as a novel mechanism influencing interaction dynamics of the TA module components.

**IMPORTANCE:**

Bacterial pathogens rely on the phenomenon of persistence as a survival strategy to combat the adverse environmental conditions encountered during infection. As a stochastic process, the driving force(s) that potentiate the formation of persisters in a bacterial population are largely unclear. This study is a step towards the discovery of intricate regulatory mechanisms that coordinate a synchronized TA cellular program. We propose a model where the TA module is regulated post-translationally, specifically via Ser/Thr phosphorylation disrupting the interaction between the toxin and antitoxin proteins as a mechanism to regulate TA function.

## INTRODUCTION

Tuberculosis (TB) originated as a devastating disease in Western Europe during the Industrial Revolution and remains a serious infectious disease accounting for 1.6 million deaths worldwide (1). The continued pathogenic success of *Mycobacterium tuberculosis*, the causative agent of TB, can be attributed to its ability to adapt and persist in host granulomas, in a non-replicating and drug-tolerant state for decades with the potential of disease reactivation in elderly or immunocompromised individuals (2, 3). While considerable progress has been achieved in TB research over the last two decades, enhancing our comprehension of host-pathogen interactions, immune responses, and shifts in metabolic processes during infection, the precise mechanisms that drive the adaptive strategies of mycobacterial growth remain elusive.

By definition, a persister cell is one that displays a remarkable ability to tolerate adverse conditions and survive exposure to antibiotics or other stress-inducing factors that would typically kill the majority of the bacterial population (4–6). Physiological heterogeneity of a bacterial population enables the formation of persisters in a probabilistic manner. Numerous studies predominantly using *Escherichia coli* as model bacteria have revealed a variety of factors and stress response pathways that seem to be connected to the development of persister cells (7–11). One such means of achieving persistence involves toxin-antitoxin (TA) modules that consist of a pair of closely linked genes encoding a toxin and an antitoxin (12, 13). The toxin component of the module is a protein that, when present in excess, interferes with essential cellular processes, resulting in growth arrest or cell death (13). Conversely, the antitoxin is a protein or RNA molecule that counteracts the toxic effects of the toxin by binding to it, sequestering it, or inhibiting its biological activity (14, 15). Based on the nature of the antitoxin, eight families of TA modules have been described (16) with type II TA modules representing the most widely studied and abundant TA family observed in prokaryotes. Typically, type II modules are composed of a protein toxin and a cognate antitoxin protein that interact via protein-protein interaction (17). While several type II systems have been evaluated in *E. coli* and *Mycobacterium smegmatis* for their cytotoxicity (18–21), others such as VapBC, MazEF, ParDE, RelBE, and HigAB have been implicated in stress-induced survival of *M. tuberculosis* (19, 22–27).

Understanding how TA expression is controlled and fine-tuned is critical to our comprehension of their role in mycobacterial physiology, fitness, and pathogenicity. In general, most TAs are autoregulated by either the antitoxin protein alone or by the toxin-antitoxin complex (12). Understandably, the relative stoichiometry of the toxin and antitoxin proteins is a crucial determinant of their interaction outcomes. It follows that the coordinated expression of TA modules and regulation of toxin-antitoxin binding and antitoxin degradation must be precise and likely dependent on multiple factors such as the binding affinities of the toxin and antitoxin, stability of small antisense RNA, and post-translational modifications (PTMs) (28–30). A recent report describing toxin neutralization by its cognate antitoxin (or atypical kinase) via phosphorylation has generated significant interest (31). Likewise, Dawson et al. demonstrated post-transcriptional regulation of the *M. tuberculosis* RelBE system (32). Given their crucial roles in cell death and persistence, we are still far from decoding how TA modules are regulated, both as independent units and as multi-family complexes.

Post-translational modification such as *O-*phosphorylation, also known as Ser/Thr/Tyr phosphorylation, is one of the most universal forms of regulating protein function by covalent modification (33). They allow the bacteria to rapidly respond to changing environments and adapt to various conditions, making them crucial for bacterial physiology and survival. The *M. tuberculosis* genome encodes 11 “eukaryotic-like” Ser/Thr protein kinases (STPKs) and three phosphatases that help regulate the extent and duration of *O*-phosphorylation (34, 35). The reversible nature of phospho-Ser/Thr/Tyr signaling, along with the wide array of diverse functions governed by *O*-phosphorylation, strengthens its role as a prominent central regulatory mechanism.

In this study, we investigated the regulation of TA modules by post-translational modification, specifically Ser/Thr phosphorylation. Given our previous studies on TA modules, we focused on the *M. tuberculosis* RelBE TA family that includes three TA loci, namely, RelBE1 (*Rv1246c–1247c*), RelBE2 (*Rv2865–Rv2866*), and RelBE3 (*Rv3357–Rv3358*). Since these were originally referred to as RelBE, RelFG, and RelJK, respectively (19), we maintained the same nomenclature in this study. All three loci have been characterized as bona fide TA modules (19) that are responsive to stress environments such as nitrogen starvation and inhibit growth by targeting translation (36). We explored a link between the Rel TA modules and PknK, a cytosolic, growth-regulatory Ser/Thr protein kinase that is, coincidentally, also induced under nitrogen starvation (37) and implicated in translational control mechanisms (38). We demonstrate that PknK interacts with RelE, RelK toxins, and the RelJ antitoxin proteins *in vitro* and in mycobacteria. LC-MS/MS analysis identified several sites in the RelJ antitoxin and one site (Thr77) in the RelK toxin as the site of PknK-mediated phosphorylation. Simulation studies suggest a role for the Thr77 residue in enabling stable toxin-antitoxin interaction such that its phosphorylation renders significant conformational changes in the RelK toxin, disrupting its interaction with the RelJ antitoxin. *In vitro* binding and co-expression studies corroborate these findings and reveal a unique regulatory mechanism to modulate RelJK function.

## RESULTS

### *In silico* prediction of post-translational modifications of *M. tuberculosis* TA modules

**Fig 1:**
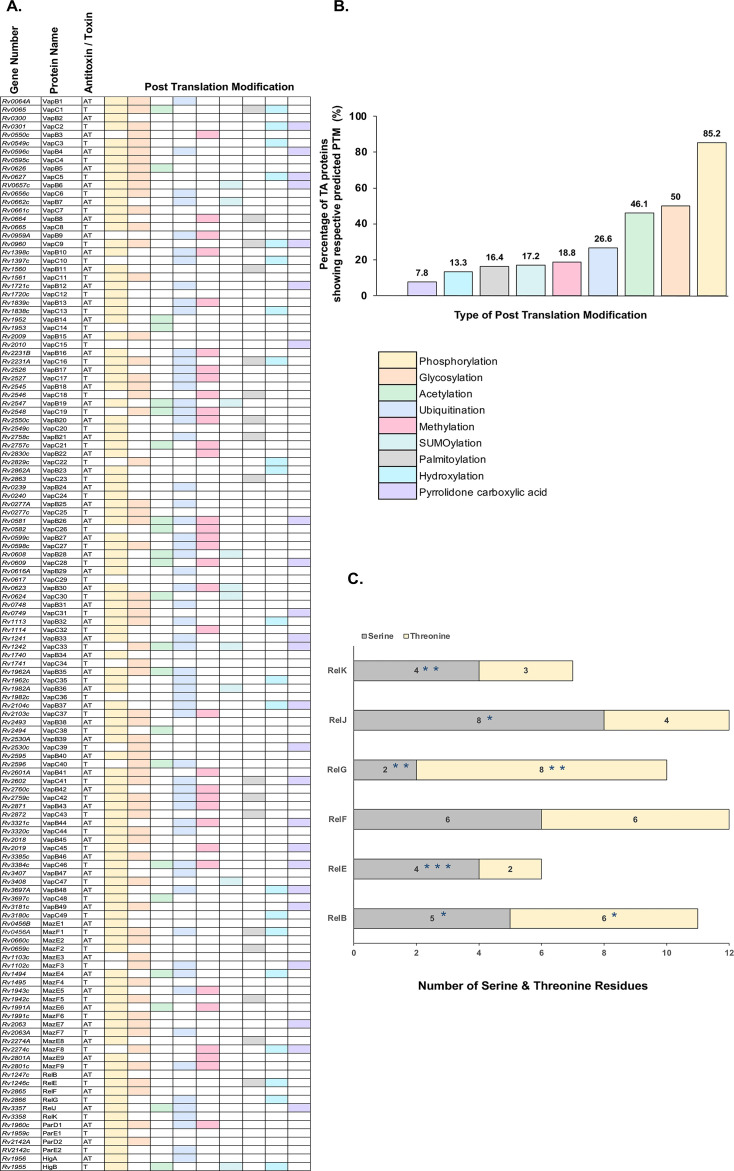
*In silico* analysis of predicted PTMs for *M. tuberculosis* TA proteins. (**A**) Distribution of nine different PTMs among the 128 *M*. *tuberculosis* antitoxin (AT) and toxin (T) genes as predicted by MusiteDeep software (https://www.musite.net/). The gene numbers and names for respective TA genes/proteins from *M. tuberculosis* H37Rv strain are listed (https://mycobrowser.epfl.ch/). (**B**) The TA proteins showing a potential PTM were grouped together, and the percentage is plotted to depict the percentage of TA proteins showing a respective PTM. (**C**) Potential Ser/Thr phosphosites in Rel TA family predicted by NetphosBac 1.0. Numbers in the stacked bar graph denote the total number of serine (gray) and threonine residues (yellow) in Rel TA proteins. Of the available Ser/Thr sites, the number of potential phosphosites predicted by the NetPhosBac 1.0 tool is marked with an asterisk (https://services.healthtech.dtu.dk/services/NetPhosBac-1.0/). A list of predicted phosphosites for the RelBE TA proteins can be found in Table S1.

We have previously utilized a bioinformatics approach to report extensive phosphorylation in mycobacterial signaling proteins (39). Using the same strategy, we investigated the potential of *M. tuberculosis* TA proteins to undergo post-translational modifications. We used MusiteDeep for PTM prediction, an online resource providing a deep-learning framework for protein post-translational modification site prediction and visualization (40–42). The predictor only uses protein sequences as input, and no complex features are needed, resulting in a realistic prediction for proteins (42). We analyzed 128 *M*. *tuberculosis* toxin and antitoxin proteins belonging to 64 paired TA modules of Vap, Maz, Rel, Par, and Hig families and grouped them in nine different PTM categories (Fig. 1A and B). Remarkably, the percentage of PTM by phosphorylation was significantly higher than other predicted PTMs (Fig. 1A and B), suggesting that phosphorylation of TA proteins may be a ubiquitous mechanism to modulate TA function. Interestingly, with the exception of three antitoxins (VapB3, VapB9, and MazE3), all antitoxins showed putative phosphosites. In contrast, 25% of the toxins analyzed were not positive for phosphorylation. For smaller TA families such as Par and Hig, all TA proteins were amenable to Ser/Thr phosphorylation. These results highlight *O*-phosphorylation of *M. tuberculosis* toxin and antitoxin proteins as a widespread modification across all TA families.

Based on these results, we subjected the RelBE TA proteins to additional screening for potential phosphosites using NetPhosBac 1.0 software. While MusiteDeep predicts PTMs, the NetPhosBac 1.0 tool is based on an artificial neural networking model that predicts bacteria-specific Ser/Thr phosphorylation (43). It should be noted that NetPhosBac considers full length of the protein irrespective of any specific domain regions and only reports phosphosites with scores >0.5. Data obtained for the RelBE TA proteins using both tools, along with overlaps observed with published data (44), are compiled in Table S1.

Figure 1C depicts the total number of serine and threonine residues for each Rel protein. Of these, the number of predicted phosphosites is marked with an asterisk. In our analysis, we observed that all members of the Rel family except RelF are likely to be modified by phosphorylation. Overall, the propensity of potential serine phosphorylation was higher than threonine modification (9 Ser vs 3 Thr). It is noteworthy that Rel toxins seem more susceptible to STPK-mediated phosphorylation as compared to Rel antitoxins (Fig. 1C). Although these analyses are largely predictive, they point toward a distinct role of *O*-phosphorylation in the regulation of RelBE TA modules.

### *M. tuberculosis* STPK PknK interacts with Rel TA proteins in mycobacteria

*M. tuberculosis* STPKs are established regulators of diverse cellular processes ranging from regulation of growth, cell division, membrane biogenesis, metabolism, and pathogenesis (45). Upregulation of *rel* and *pknK* transcripts under conditions of nitrogen starvation (36, 37) and their independent roles in translational control (36, 38) prompted us to investigate PknK-mediated phosphorylation of the *M. tuberculosis* Rel TA proteins.

**Fig 2:**
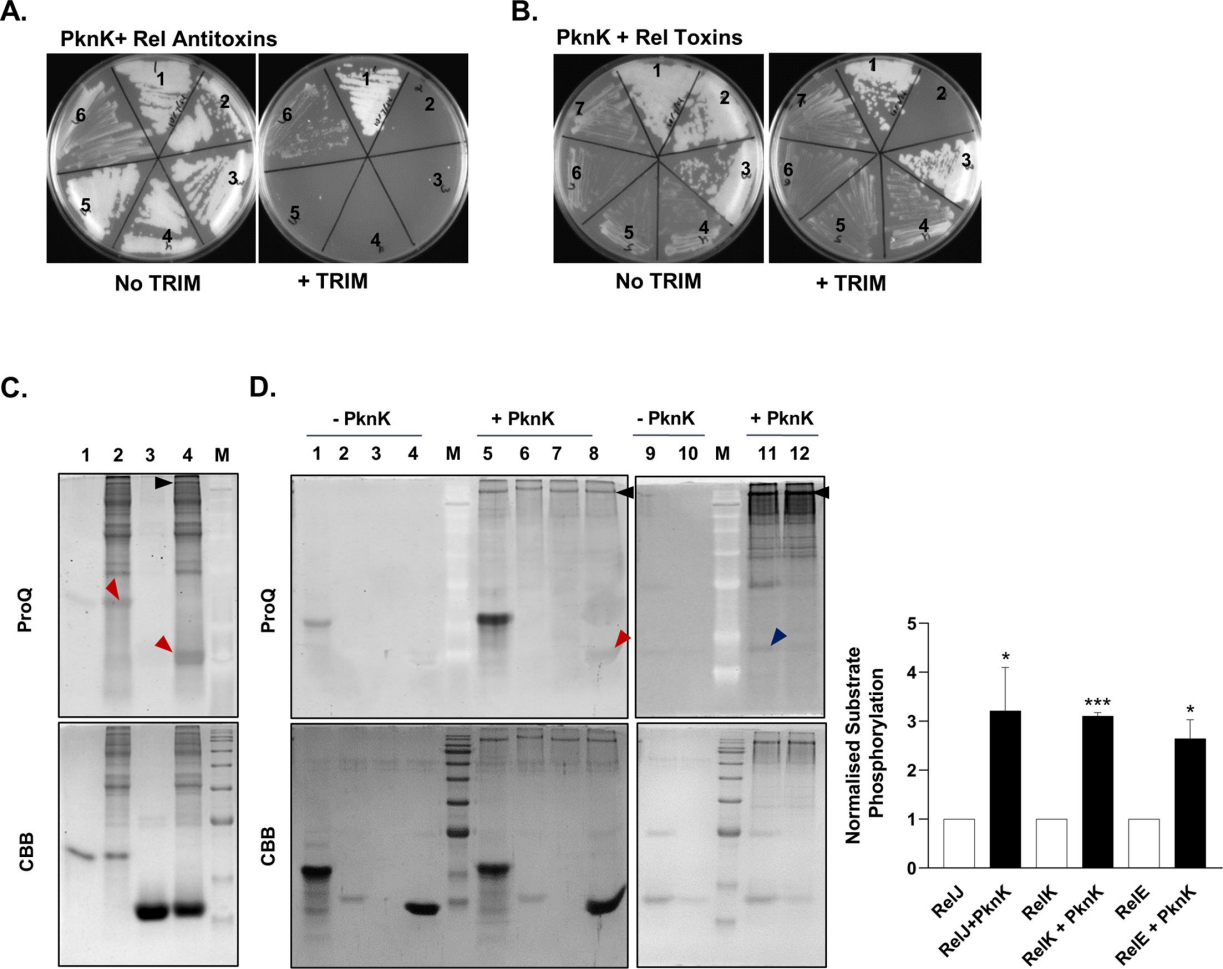
Rel TA proteins interact with PknK *in vitro* and in mycobacteria. M-PFC assay depicting protein interactions in mycobacterial cells between PknK and (**A**) Rel Antitoxins. *M. smegmatis* was co-transformed with positive control pUAB100 + pUAB200 (section 1), negative control RelB_[F1,2]-C_/pUAB200 (section 2), RelB_[F1,2]-N_/PknK_[F3]-N_ (section 3), RelB_[F1,2]-C_/PknK_[F3]-N_ (section 4), RelF_[F1,2]-N_/PknK_[F3]-N_ (section 5), RelJ_[F1,2]-N_/PknK_[F3]-N_ (section 6), and with (**B**) Rel toxins. *M. smegmatis* was co-transformed with positive control pUAB100 + pUAB200 (section 1), negative control RelE_[F3]-C_/pUAB300 (section 2), RelB_[F1,2]-N_/RelE_[F3]-N_ (section 3), RelE_[F3]-N_/PknK_[F1,2]-N_ (section 4), RelE_[F3]-C_/PknK_[F1,2]-N_ (section 5), RelG_[F3]-N_/PknK_[F1,2]-N_ (section 6), and RelK_[F3]-N_/PknK_[F1,2]-N_ (section 7). Transformants were streaked on 7H11-Kan-Hyg plates and on 7H11-Kan-Hyg-TRIM plates to select for protein-protein interactions. Two independent experiments were performed, and one representative picture is presented. (**C**) *In vitro* kinase assay of wild-type PknK with RelJ Antitoxin. All gels are stained with ProQ Diamond phosphostain on the top panel and CBB on the bottom panel. Myelin basic protein (MBP) was used as a positive control. Lanes 1 and 2 show MBP without and with PknK, respectively. Lanes 3 and 4 show RelJ alone and with PknK, respectively. Red arrow on the top gel panel shows the phosphorylation of MBP (lane 2) and RelJ (lane 4) by PknK. (**D**) Left: *in vitro* kinase assay of PknK with Rel Toxins (RelE, RelG, and RelK). Lanes 1 and 5 show MBP without and with PknK, respectively. RelE, RelG, and RelK alone (lanes 2–4) and with PknK (lanes 6–8). Red arrow indicates the phosphorylation of RelK by PknK (lane 8). Kinase assay of RelE and RelG with PknK (lanes 11 and 12). Blue arrow depicts the phosphorylation of RelE by PknK (lane 11). Black arrows indicate auto-phosphorylated PknK. Right: fold change increase in signal intensity of RelJ, RelK, and RelE in the presence of PknK is plotted as bar graphs (solid black bars) relative to the unphosphorylated controls (solid white bar; baseline set at 1.0). Data are plotted as mean ± SD from three independent replicate gels, and one representative gel is presented. * and *** represent *P* < 0.05 and 0.001, respectively, for the difference in fold intensities of the Rel protein observed in the presence of the kinase vs unphosphorylated control.

The Mycobacterial Protein Fragment Complementation (M-PFC) assay (46) was used to detect PknK interactions with either Rel toxin or antitoxin proteins *in vivo* using the model organism *M. smegmatis* as the host (46). Previous results suggest that RelB and RelE directly bind, with interaction likely occurring between the C-termini of both proteins (19). Additionally, Korch et al. observed that the toxicity of RelE was dependent upon free C-termini, as RelE C-terminal fusion proteins were incapable of inhibiting mycobacterial growth, even in the absence of RelB. Directed by these results, we tested whether Rel antitoxin-_[F1,2]-N_ fusions or Rel toxin-_[F3]-N_ fusions interact with PknK-_[F1,2]-N_ or PknK-_[F3]-N_ fusion proteins in *M. smegmatis*. Controls included *M. smegmatis* co-transformed with the empty vectors pUAB100/pUAB200 (GCN4_[F1,2]_/GCN4_[F3]_) as positive control and the empty vector pUAB200/RelB_[F1,2]-N_ or with the empty vector pUAB300/RelE_[F3]-C_ as negative controls. As seen in Fig. 2A and B, all antitoxin- or toxin-PknK co-transformations resulted in growth; however, the growth of RelE/G/K-PknK co-transformants was less robust, likely due to the toxin activity in the absence of the antitoxin (Fig. 2B), an observation that is in agreement with an earlier study (19). Furthermore, when grown in the presence of trimethoprim (TRIM) and thus requiring protein interaction for survival, we only observed reproducible growth of *M. smegmatis* for the following protein pairs: RelJ_[F1,2]-N_/PknK_[F3]-N_, RelE_[F3]-N_/PknK_[F1,2]-N_, and RelK_[F3]-N_/PknK_[F1,2]-N_ and the positive controls GCN4_[F1,2]_/GCN4_[F3]_ and RelB_[F1,2]-N_/RelE_[F3]-N_ (Fig. 2A and B). These results indicate that PknK selectively interacts with RelE and RelK toxins and the RelJ antitoxin protein in mycobacteria.

### Rel toxin and antitoxin proteins are differentially phosphorylated by PknK

To further characterize the STPK-Rel TA interactions, recombinant Rel proteins were purified and subjected to *in vitro* kinase assays with the wild-type or phosphorylation-defective PknK. Autophosphorylation and transphosphorylation activities of PknK were confirmed using myelin basic protein (MBP) as a general kinase substrate, which also served as a positive control in our assays (Fig. S1).

For kinase assays, positive reactions were visualized by the ProQ Diamond Phosphoprotein Gel Stain that specifically stains phosphorylated S/T/Y amino acids (47). These assays were performed in triplicates, and one representative gel each for antitoxin and toxin proteins is shown in Fig. 2C and D, respectively. The Rel proteins were tested for basal levels of background phosphostaining in the absence of the kinase. While no signal was observed with RelB and RelF antitoxins (Fig. S2A), both MBP and RelJ showed a change in the signal intensities in the presence of PknK as compared to proteins alone in ProQ-stained gels (Fig. 2C; lanes 2 and 4 vs lanes 1 and 3). Similarly, with respect to the toxin proteins, a change in phosphosignal intensity was observed for RelK in the presence of PknK as compared to RelK alone (Fig. 2D; compare lanes 4 and 8). These observations suggest that the cognate TA pair, RelJ and RelK, are substrates for PknK-mediated phosphorylation. There was no phosphosignal observed for RelE and RelG proteins (Fig. 2D; lanes 6 and 7). We noticed that the Rel toxins were susceptible to aggregation. Despite equal loading, the protein amounts observed in the Coomassie Brilliant Blue R250 (CBB)-stained gel were considerably lower for RelE and RelG than RelK (Fig. 2D; lanes 2 and 3, lower panel). Thus, we repeated assays with fresh preparations of RelE and RelG (Fig. 2D; right) and observed a faint signal for phosphoprotein staining with RelE in the presence of PknK and ATP (Fig. 2D; compare lanes 9 and 11). There was no observable difference in signal intensities obtained with the RelG toxin. Results were consistent across replicate gels, and thus, densitometric analysis was done for all replicates to calculate fold change as described in Materials and Methods. The graph shown in Fig. 2D (right panel) shows a ~3.2-fold, ~3.1-fold, and ~2.6-fold increase in the intensity of the phosphostained RelJ, RelK, and RelE substrate proteins, respectively, in the presence of PknK as compared to the substrate alone. It may be noted that the intensity of the phosphosignal corresponds to the number of residues phosphorylated in a given protein.

To confirm that the phosphorylation signal observed for RelE, RelK, and RelJ is PknK-specific, we performed the same assay in the presence of phosphorylation-defective PknK K55M mutant protein. As expected, there was no increase in the signal observed for RelE, RelK, or RelJ substrate proteins when incubated with the mutant PknK protein (Fig. S2B). Based on these results, we concluded that toxins RelE and RelK and the antitoxin RelJ are targets for phosphorylation by PknK.

### Kinetics of PknK-mediated phosphorylation of Rel TA proteins

**Fig 3:**
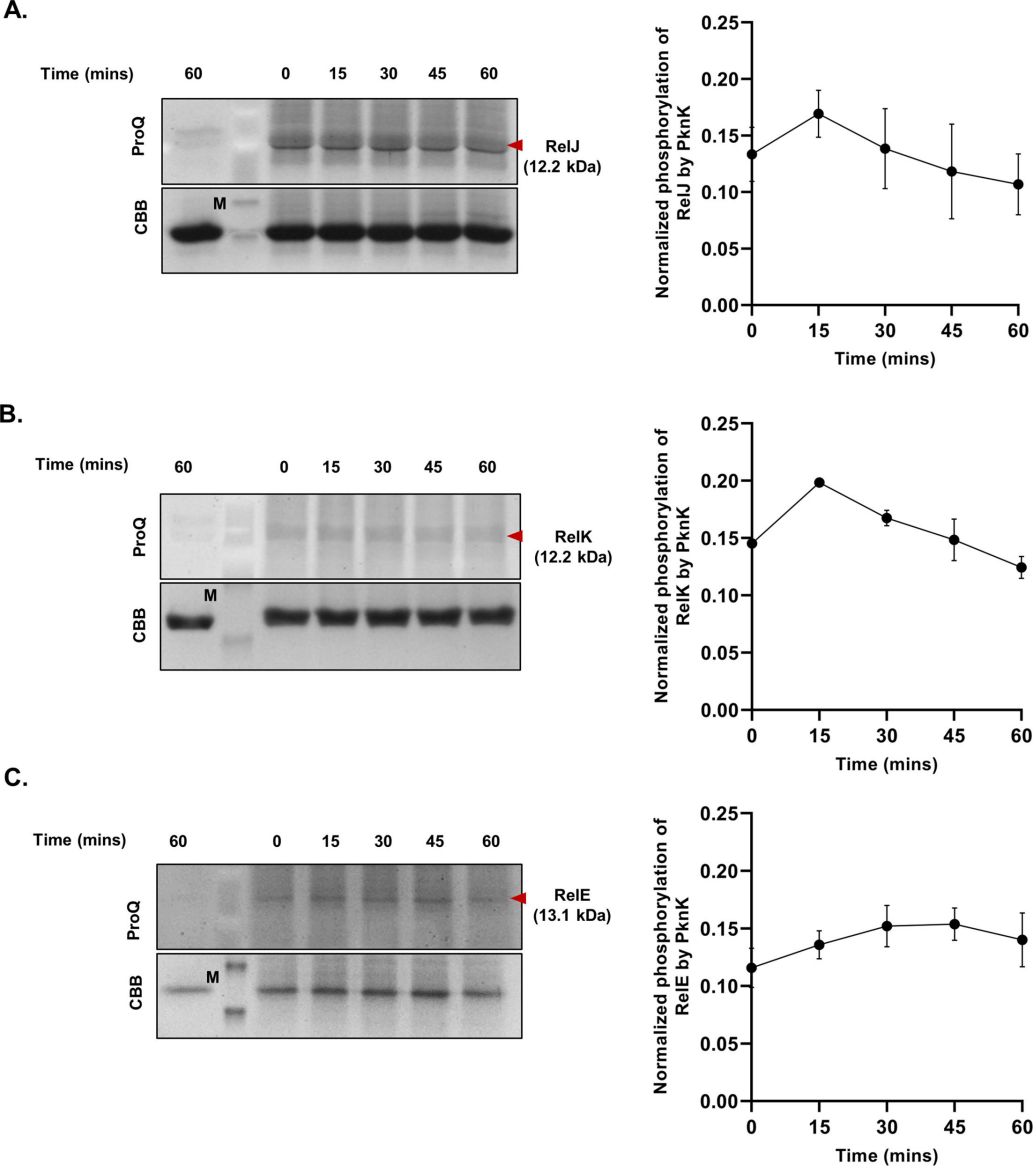
Time kinetics of PknK-mediated phosphorylation of Rel TA proteins. (**A**) RelJ antitoxin. (**B**) RelK toxin. (**C**) RelE toxin. In all images, lane 1 shows the substrate proteins alone. The time course was done at the indicated time points with PknK and substrate, i.e., antitoxin or toxin (indicated by red arrows). All gels are stained with ProQ Diamond on the top panel and CBB on the bottom panel. One representative gel picture is shown, and a trend of relative phosphorylation over time is reported. Data are plotted as mean ± SD from three independent experiments.

To perform time kinetic analysis for phosphorylation of RelE, RelJ, and RelK, proteins were incubated alone or with PknK for 0, 15, 30, 45, and 60 min, and relative phosphorylation levels were calculated from three independent assays using the ImageJ software (Fig. 3). The trend of phosphorylation was similar for RelJ and RelK proteins, attaining a maximum signal at 15 min, followed by a consistent but insignificant decrease in the signal by 60 min (Fig. 3A and B). RelE displayed a distinctively different pattern with a maximum phosphosignal observed at the 30 and 45 min time point, after which there was a slight decrease in the signal intensity over 60 min (Fig. 3C). Typical of Ser/Thr phosphorylation, the phosphosignal on RelE, RelJ, and RelK was found to be stable up to 24 h post-phosphorylation (data not shown).

### Identification of RelJK phosphosites by mass spectrometry

**Fig 4:**
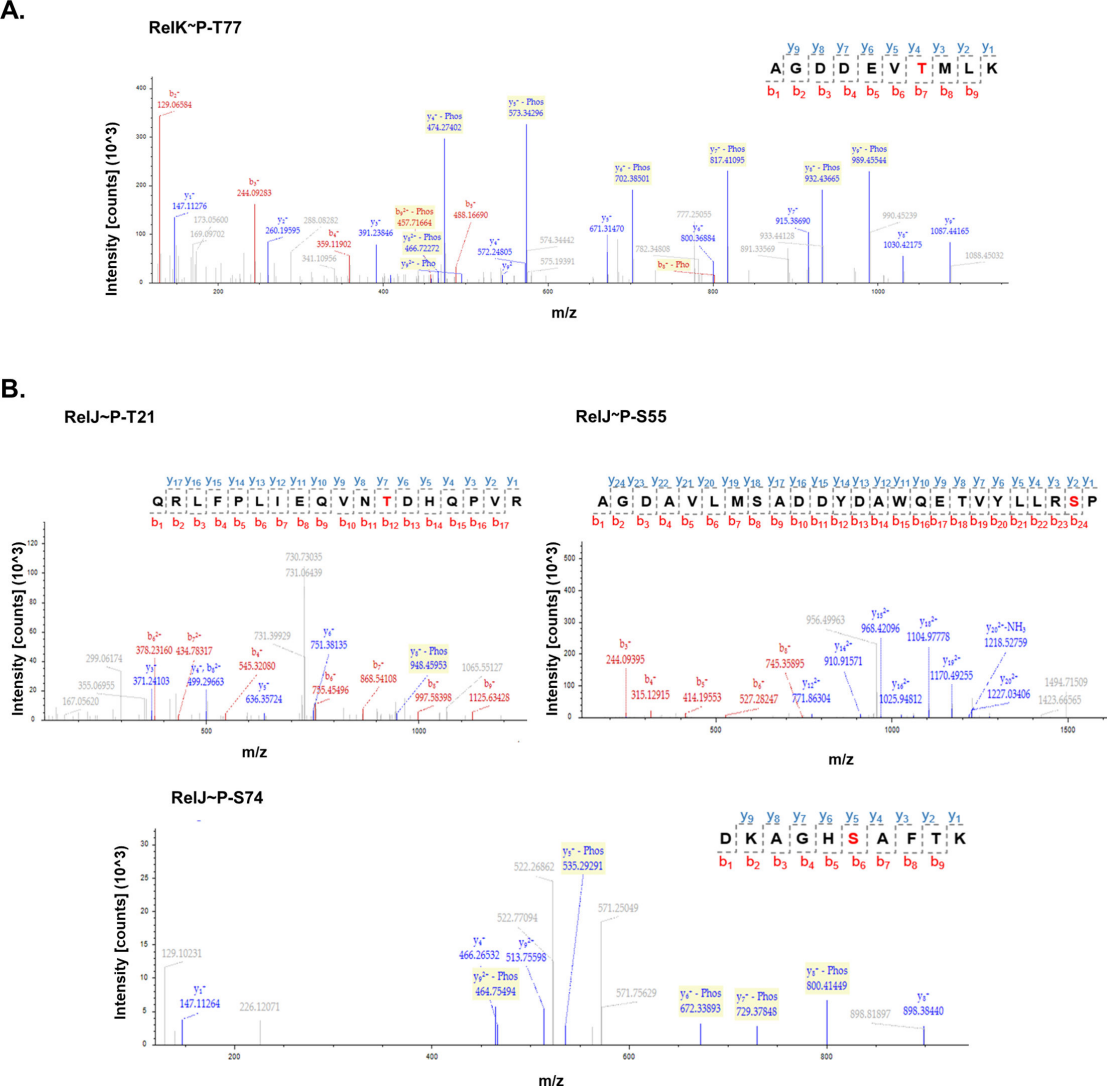
LC-MS/MS analysis for identification of phosphorylated residues. Mass spectra analysis for (**A**) RelK toxin and (**B**) RelJ antitoxin proteins. The respective peptides with the phosphorylated residue (highlighted in red) are shown in the inset. Peptide spectrum match and protein false discovery rate (FDR) were set to 0.01 FDR to obtain high confidence hits.

To identify the specific amino acid phosphorylated by PknK, the phosphorylated TA proteins were subjected to LC-MS/MS analysis. Multiple high-confidence phosphosites were identified for the RelJK TA proteins. As seen in Fig. 4, MS spectra revealed a single phosphorylated site in RelK, identified as threonine at position 77 (Fig. 4A). Additionally, several PknK-dependent phosphorylation sites were identified in RelJ antitoxin at amino acid positions 21 (Thr), 55 (Ser), and 74 (Ser) of the RelJ polypeptide chain (Fig. 4B). Details of the LC-MS/MS results are available in Tables S2 and S3.

### Thr77 phosphorylation is a crucial determinant of RelK secondary structure

**Fig 5:**
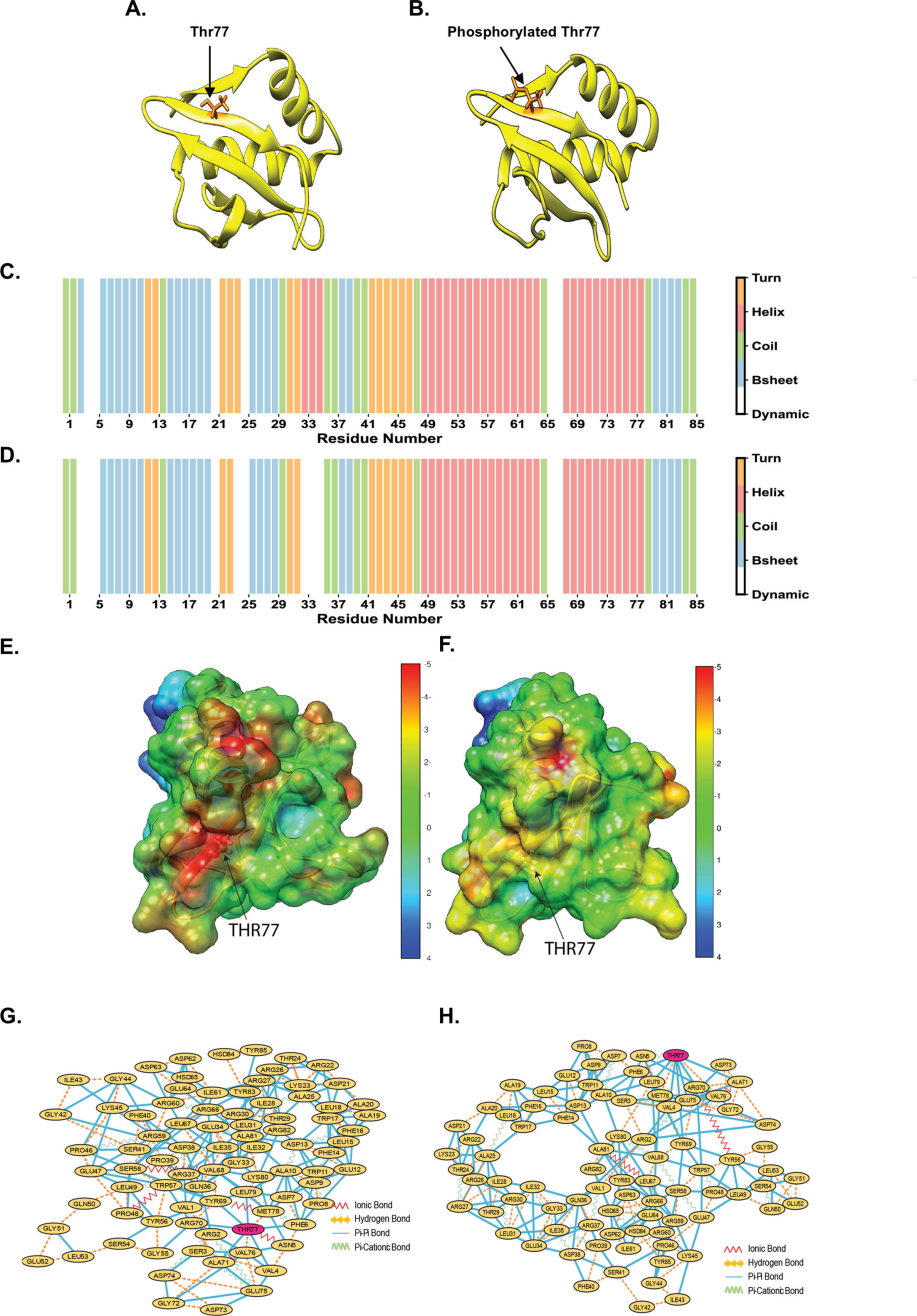
Structural changes associated with phosphorylation of RelK toxin. (**A**) Structure of RelK toxin (control) and (**B**) structure of RelK-phosphorylated at Thr77 residue (RelK~P). Residue and domain-wise assignment of the secondary structure among turn (yellow), helix (peach), coil (green), β-sheet (blue), and dynamic residues (white) of (**C**) RelK and (**D**) RelK~P. The secondary structure assignment is based on the propensity of a residue to exist in a secondary structure for more than 75% of the simulation time. Residues that did not conform to a single secondary structure for >75% of the simulation length were considered to be dynamic. Electrostatic potential map showing phosphorylation-induced changes at Thr77. (**E**) RelK exhibits a highly negative electrostatic potential (−5 kT/e, red). (**F**) RelK~P (phosphorylated) shows a shift to a moderately negative potential (−3 to −4 kT/e, yellow), indicating charge redistribution. This reduction in negative potential may weaken electrostatic interactions within the complex, potentially decreasing binding affinity and influencing protein stability and function. (**G and H**) Cytoscape image of the residue interaction network. Circles in pink depict the Thr77 residue.

Post identification of the solitary site of phosphorylation (Thr77) with RelK toxin, we focused on the biophysical characterization of RelK’s PTM. Molecular dynamics simulations (MDSs) were carried out on individual RelK toxin (control) and RelK-phosphorylated at Thr77 residue (henceforth referred to as RelK~P) to study the dynamic behavior of the RelK toxin in the presence of the PTM (Fig. 5). Quality check parameters for the simulated system (temperature, pressure, and density) were evaluated to check the validity of the performed simulations (Fig. S3).

The root mean square deviation (RMSD) results revealed that the RelK unphosphorylated control reached a stable RMSD of 0.19 nm in 175 ns, while the RelK~P chain reached a stable RMSD of 0.20 nm in 200 ns, suggesting that the equilibration of the toxin chain varied based on its phosphorylation state (Fig. S3C). Similar results were obtained when we analyzed the compactness of the protein radius of gyration (Rg) for unphosphorylated RelK control and phosphorylated RelK~P chains (Fig. S3D). Root mean square fluctuation (RMSF) was assessed to determine the impact of phosphorylation on fluctuations of the chain residues during the simulation. The RMSF values per residue for the RelK~P chain showed a slight increase to 0.1306 Å compared to the unphosphorylated RelK control with a state of 0.0787 Å (Fig. S3E). This suggests that there is an elevation in the motion of residues within RelK~P as compared to the protein in its unphosphorylated state.

Upon investigating the changes in the secondary structure, we observed significantly more pronounced fluctuations in the RelK~P chain as compared to the control chain. More importantly, we noted a decline in β-sheet and α-helix secondary structures in the RelK chain due to phosphorylation at Thr77 during the 250 ns simulation. Specifically, three α-helices and one β-sheet were lost in the RelK~P chain (Fig. 5C and D). The distinct loss of secondary structure elements indicates that phosphorylation imparts long-distance perturbations rather than being restricted to the phosphosite’s immediate vicinity.

To further explore the structural and electrostatic consequences of phosphorylation, we performed principal component analysis (PCA) and electrostatic potential mapping using the Adaptive Poisson-Boltzmann Solver (APBS). PCA revealed a significant shift in dominant motion patterns, indicating an increase in global protein flexibility and a redistribution of large-scale conformational dynamics in RelK~P compared to RelK (Fig. S4). An animation movie illustrating the structural rearrangements described above is included in the supplementary material (Fig. S4). The APBS electrostatic potential map (Fig. 5E and F) further highlighted that phosphorylation at Thr77 alters charge distribution, shifting from highly negative (−5 kT/e, red) to moderately negative (−3 to −4 kT/e, yellow). This redistribution likely weakens electrostatic interactions and may contribute to the observed structural instability in RelK~P.

The long-range perturbation guided us towards exploring alterations in the residue interaction network following phosphorylation at Thr77. Fig. 5G and H illustrate the disruption of an ionic bond at Thr77 post-phosphorylation. Moreover, extending the DSSP plot findings, which depict secondary structure changes around Gly33, Cytoscape analysis further revealed the loss of Pi-Pi interactions between Gly33 and residues Ile32, Lys80, Leu67, and Ile35. This loss correlates with the disruption of α-helix structures at these sites in RelK~P compared to the unphosphorylated RelK chain (Fig. 5G and H).

Together, these results from secondary structure analysis, PCA, APBS, and residue interaction mapping indicate that phosphorylation at Thr77 induces a conformational shift associated with secondary structure deviation and intra-chain residue network rewiring. This led us to predict that such changes may impact the interaction of the RelK toxin with RelJ, its cognate antitoxin.

### Molecular dynamic simulations reveal a destabilized RelJ-RelK~P complex

**Fig 6:**
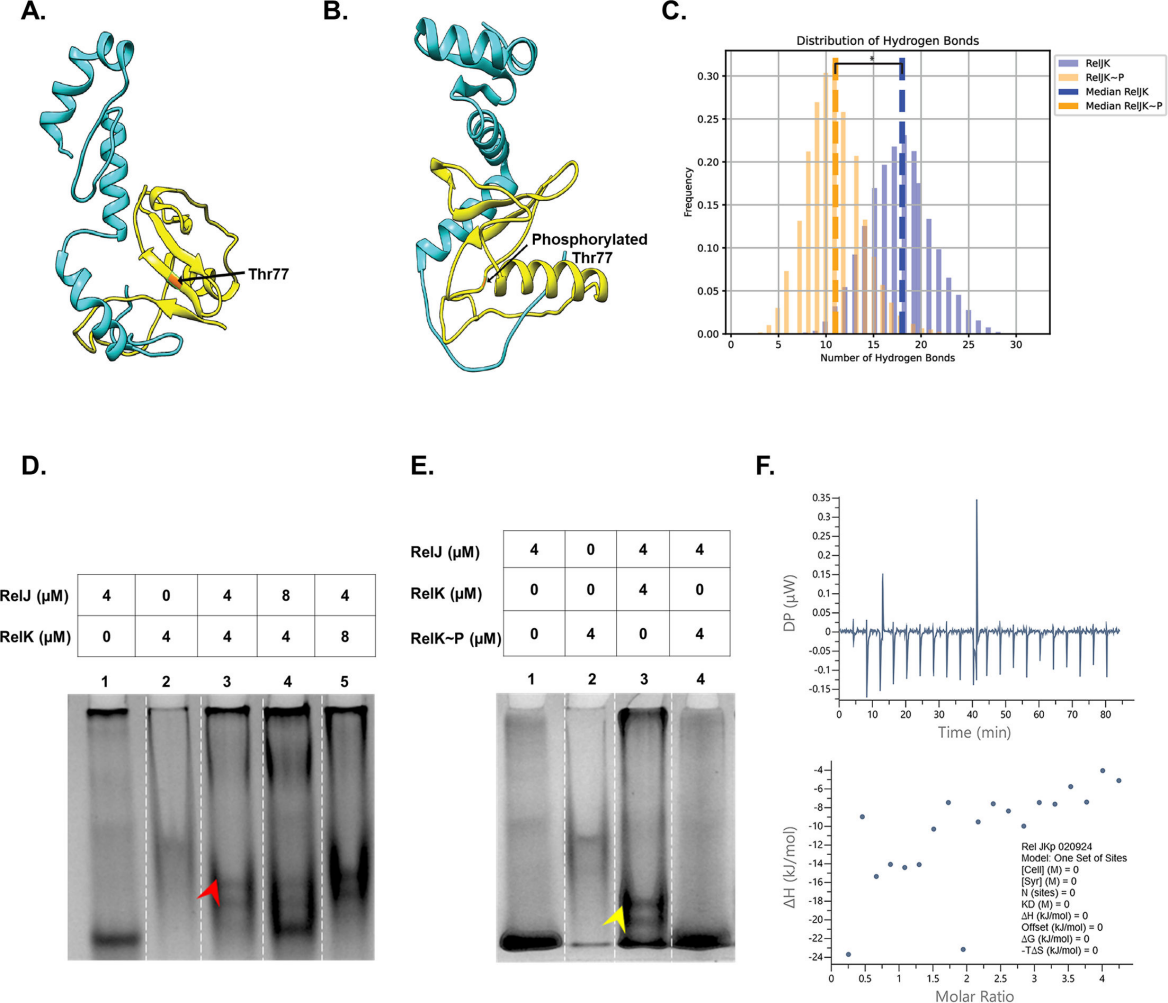
Phosphorylation of RelK at Thr77 interferes with its binding to RelJ antitoxin. (**A**) Structure of RelJK complex (RelJ in blue and RelK in yellow, unphosphorylated control) showing Thr77 residue in orange. (**B**) Structure of RelJK~P (RelK phosphorylated at Thr77) showing phosphorylated Thr77. (**C**) The plot depicts the hydrogen bond frequency distributions for “RelJK” in blue and “RelJK~P” in orange along the duration of MDS. Dashed lines represent the medians for the number of hydrogen bonds formed between RelJ and RelK in the RelJK and RelJK~P complexes. * Represents *P* < 0.05 for the difference in the medians observed for RelJK and RelJK~P complexes. (**D**) Interaction of RelJ with RelK protein on 8% Native PAGE. Lanes 1 and 2 are RelJ and RelK alone, respectively. RelJ was incubated with RelK in 1:1, 2:1, and 1:2 ratios in (lanes 3, 4, and 5), respectively. Red arrow indicates the shift observed upon binding of wild-type RelJ and RelK proteins. (**E**) Effect of RelK phosphorylation on RelJK interaction. RelK was phosphorylated by PknK and purified as described in Materials and Methods. Lanes 1 and 2 are RelJ and RelK proteins alone, respectively. RelJ was incubated with unphosphorylated RelK (lane 3) and with purified RelK~P (lane 4). Yellow arrow indicates the shift observed upon binding of RelJ with unphosphorylated RelK protein that is absent with the RelK~P protein (lane 4). The binding assays were performed in duplicate, and one representative gel is shown. (**F**) Isothermal titration calorimetry with RelJ and RelK~P proteins. No binding was observed, suggesting that phosphorylation of Thr77 interferes with the binding of RelK to RelJ antitoxin.

Guided by our results, we sought to investigate how phosphorylation of RelK affects its interaction with cognate antitoxin RelJ. MDSs were carried out on individual, unphosphorylated RelJ chain and toxin-antitoxin complexes in the following combination: RelJK (unphosphorylated control; Fig. 6A) and RelJ (unphosphorylated) with RelK phosphorylated at Thr77 (henceforth referred to as RelJK~P; Fig. 6B) to study the dynamic behavior of the toxin-antitoxin interactions, with and without the PTM. As done previously, the systems were stabilized, and no significant alterations in density, temperature, and pressure were observed in the RelJ chain (Fig. S5) and the RelJK complexes during the MDS period (Fig. S6). RMSD calculations revealed that the control RelJK complex stabilizes after 125 ns at 1 nm, while the RelJK~P complex stabilizes after 175 ns at 0.8 nm (Fig. S6C). Furthermore, the radius of gyration for the control-RelJK complex was found to stabilize after 125 ns at 2 nm, while the phosphorylated RelJK~P complex stabilized after 140 ns at 1.87 nm (Fig. S6D).

Since earlier results indicated a dramatic change in the RelK secondary structure due to Thr77 phosphorylation, we assessed hydrogen bond formation in the TA complexes with and without the RelK-PTM. A pronounced effect of the phosphorylation was seen in the antitoxin-toxin hydrogen bonding network (Fig. S6E). The figure clearly highlights significant alterations in the global protein arrangement. As shown in Fig. 6C, approximately 18 hydrogen bonds are formed between RelJ and RelK chains in the control-unphosphorylated RelJK complex, while only 11 bonds were observed between the RelJ and RelK~P chains of the RelJK~P complex. Statistical analysis revealed that the observed difference in the medians of hydrogen bonds formed in the two complexes is significant (Fig. 6C). This is indicative of a destabilized toxin-antitoxin complex. Furthermore, protein-protein binding affinity between RelJ and RelK proteins, with and without Thr77 PTM, was calculated using PPI-affinity (48). Results indicate stronger interactions between RelJ and RelK in their unphosphorylated state (RelJK) having a binding affinity of −11.7 kcal/mol compared to the phosphorylated state (RelJK~P) where the binding affinity was determined to be −9.6 kcal/mol. These observations reiterate that phosphorylation attenuates the strength of RelJ and RelK protein-protein interaction.

### Phosphorylation of RelK toxin interferes with its binding to the RelJ antitoxin

To validate the computational results and to visualize protein-protein binding, we incubated unphosphorylated RelJK proteins for 30 min at 30°C and resolved the complexes on Native PAGE (Fig. 6D). Given that RelJ antitoxin binds a single molecule of RelK toxin, with the RelJK dimer interacting through association between antitoxins to form a heterotetrameric complex (49), we incubated RelJ and RelK proteins in 1:1, 2:1, and 1:2 molar ratios as indicated in Fig. 6D. A significant shift in the mobility of RelJ was observed when it was incubated with RelK in equimolar ratio (Fig. 6D, lane 3). Notably, the majority of RelJ was observed in a bound state, as demonstrated by the shift in molecular size. Furthermore, a marginal amount of RelJ was observed in the unbound state when the proteins were incubated in a molar ratio of 2:1 (Fig. 6D, lane 4). By contrast, the shift was more pronounced when RelK toxin was doubled as compared to RelJ (Fig. 6D, lane 5), indicating that the proteins may form oligomeric complexes with different stoichiometries.

Next, we compared RelJK complex formation in unphosphorylated and phosphorylated states. For these experiments, RelK protein was phosphorylated *in vitro* and further purified as described in Materials and Methods. We observed reproducible shifts in RelJ mobility when unphosphorylated RelJK proteins were co-incubated (Fig. 6E, lane 3). Interestingly, no shift was observed when RelJ was incubated with RelK~P protein (Fig. 6E, lane 4). These data clearly suggest that phosphorylation of RelK interferes with the RelJK~P complex formation. Finally, we confirmed the lack of interaction between RelJ and RelK~P proteins using isothermal titration calorimetry (Fig. 6F). Cumulatively, the results presented in Fig. 5 and 6 establish toxin phosphorylation as a means to interfere with toxin-antitoxin interaction.

### Thr77 residue is required for interaction with RelJ antitoxin and rescue

To confirm our findings, we replaced the Thr77 residue in RelK with alanine (T77A) to create a RelK_Mut_T77A mutant protein that was phosphodeficient (Table S4). Native PAGE analysis implicated Thr77 at the binding interface between RelK and RelJ proteins (Fig. S7). To understand the effect of toxin phosphorylation in cells, we created a phosphomimetic mutant where Thr77 was replaced with glutamate (T77E).

**Fig 7:**
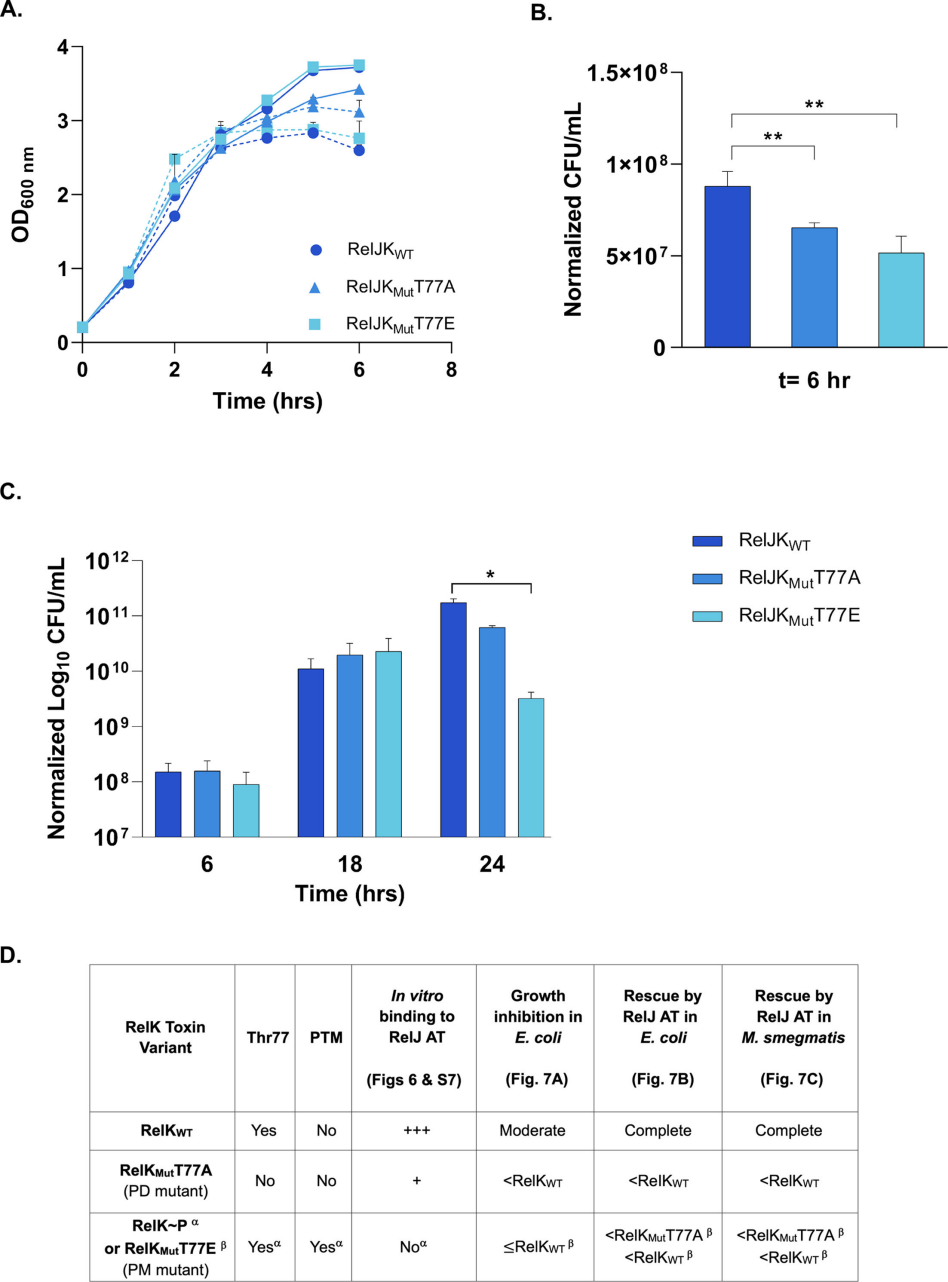
Rescue of RelK toxin variants in *E. coli* and *M. smegmatis.* (**A**) Plot of OD_600 nm_ vs time showing the growth inhibitory effect of RelK toxin variants in *E. coli* cells co-transformed with plasmid vectors, pLTA(*relJ*)20 and pCAK(*relK* or *relK*T77A or *relK*T77E)10. The co-transformed cells were induced for toxin expression with 0.2% Ara (dashed lines), and growth was compared to uninduced cells (solid lines). (**B**) The effect of toxin in *E. coli* was rescued with co-expression of the RelJ antitoxin in the co-transformed cells. Viable counts were determined for each pair post-induction with ATc and Ara at the 6 h time point: RelJK_WT_, RelJK_Mut_T77A, and RelJK_Mut_T77E. The decrease in the viability of RelJK_Mut_T77A and RelJK_Mut_T77E co-expressing cells indicates inefficient neutralization of the mutant toxins by the RelJ antitoxin. ** Represents *P* < 0.01 for the difference in viable counts obtained with cells co-expressing RelJK_Mut_T77A or RelK_Mut_T77E with respect to cells co-expressing RelJK_WT_ proteins. (**C**) Rescue in *M. smegmatis* strains co-expressing wild-type RelJ and RelK (wild- type and mutant) proteins at 6, 18, and 24 h post-induction with acetamide. CFU values obtained with induced cells were normalized with respect to their uninduced controls at respective time points. Data from all growth experiments are plotted as mean ± SD from two independent biological replicates each having two technical replicates. * Denotes a *P* < 0.05 for the difference in viable counts observed between the RelJK_WT_ and RelJK_Mut_T77E at the 24 h time point, indicating partial rescue of the RelK_Mut_T77E toxin by the RelJ antitoxin in mycobacteria. (**D**) Table summarizing the results and interpretations of Fig. 6 and 7; Fig. S7, highlighting the role of RelK Thr77 residue and its phosphorylation on interaction with the RelJ antitoxin and neutralization in *E. coli* and *M. smegmatis*. ^α^ and ^β^ denote RelK~P and RelK_Mut_T77E, respectively. PD and PM refer to phosphodeficient and phosphomimetic RelK mutant proteins. AT is antitoxin.

We co-transformed *E. coli* BL21 (DE3) cells with plasmid pairs; pLTA(*relJ*)20 and pCAK(*relK* or *relK*T77A or *relK*T77E)10 to co-express the antitoxin and toxin variants induced by anhydrotetracycline (ATc) and arabinose (Ara), respectively. To study the effect of RelK toxins on the growth of *E. coli*, the co-transformants were grown in the presence and absence of the inducer Ara for 6 h, as described previously (18). The uninduced cells did not exhibit any defect in growth, suggesting insignificant leaky expression of the toxin (Fig. 7A). In agreement with published data (18, 49), RelK overexpression resulted in a modest decrease in optical density (Fig. 7A). Data from two replicative experiments revealed that overexpression of the wild-type and mutant toxins (+Ara) exhibited similar growth trends, with the difference in optical densities among the three RelK variants being statistically insignificant (Fig. 7A). These results indicate that phosphorylation or its lack thereof has no bearing on the growth inhibitory activity of RelK toxin.

For rescue studies, we induced the co-transformants with anhydrotetracycline (+ATc) and arabinose (+Ara) as described previously (18). Since the maximal growth inhibitory effect of RelK toxin was seen at 6 h post-induction (Fig. 7A), we investigated the effect on rescue at the same time point. As shown in Fig. 7B, *E. coli* cells co-expressing wild-type RelJ with RelK_Mut_T77A or RelK_Mut_T77E showed decreased viable counts compared to cells co-expressing wild-type RelJ-RelK proteins, suggesting a rescue defect with mutant RelK proteins.

Next, we investigated the rescue of wild-type and mutant RelK toxin proteins in mycobacteria. Since *M. tuberculosis relJ* and *relK* genes are co-transcribed (19) and RelK and RelJ interact in a 1:1 ratio (Fig. 6D, lane 3), we endeavored to ensure an almost equal number of toxin and antitoxin molecules expressed through a single copy of the *relJK* open reading frames (ORFs) in the genome as opposed to the two-plasmid co-expression system where the copy number differences of the two episomal plasmids overexpressing the toxin and antitoxin genes can skew the molar ratios. Towards this, we constructed *M. smegmatis* strains carrying a single integrative copy of *relJK or relJK*_Mut_T77A or *relJK*_Mut_T77E gene pairs co-expressing the RelJ antitoxin along with the wild-type or phosphodeficient (T77A) or phosphomimetic (T77E) RelK mutant protein from an acetamide-inducible promoter. Viable counts were determined at 6, 18, and 24 h post-induction in two independent replicate experiments, each having two technical replicates. We noted a similar pattern for rescue in mycobacteria as in *E. coli* (Fig. 7C). Cells co-expressing the wild-type *relJK* genes exhibited robust growth at all time points, confirming that RelJ antitoxin interacts with the RelK toxin and neutralizes any effect of the toxin. As shown in Fig. 7C, there was no significant defect in rescue at 6 and 18 h time points; however, at 24 h post-induction, cells co-expressing the RelJ antitoxin and phosphomimetic RelK_Mut_T77E toxin exhibited the least number of viable counts as compared to the wild-type RelJK co-expressing cells. The table in Fig. 7D represents collective data from Fig. 6 and 7; Fig. S7 to establish two things: first, the Thr77 residue plays a key role at the RelJ and RelK interaction interface, and second, its phosphorylation disrupts binding to the antitoxin, thereby affecting toxin neutralization.

## DISCUSSION

Emerging evidence has implicated the role of the toxin-antitoxin modules in the initiation, development, and establishment of the persistence phenotype in *M. tuberculosis.* Although functional characterization of multiple type II TA systems has been reported, there is little evidence to describe the underlying mechanisms that regulate the coordinated expression of mycobacterial TA systems. In this study, we have investigated the role of PTMs in the regulation of *M. tuberculosis* type II TA modules. Our findings suggest that a majority of Type II TA modules are potential targets of *M. tuberculosis* Ser/Thr protein kinases. Furthermore, we demonstrate and establish that *O*-phosphorylation of a Type II Rel toxin is a distinct and novel mode of regulating toxin-antitoxin interaction.

Protein post-translational modifications are ubiquitous events that have important functional consequences such as altering enzyme activity, inhibiting DNA binding activity, or altering protein conformation (50). PTMs promise to offer exquisite control of the enzymatic activities, which, in the case of TA modules and their established roles in mycobacterial persistence, is crucial to avoid spurious initiation of persister formation. So far, very little is known about the PTM of TA modules, thus understanding the role of PTMs in the regulation of TA function is of significant interest. *In silico* analyses revealed that TA modules are amenable to distinctly different post-translational modifications, with phosphorylation leading the list followed by glycosylation. Recently, Yu et al. reported a mechanism of toxin neutralization via phosphorylation (31). TakA-TglT encoded by *Rv1044-Rv1045* constitutes a unique TA module where the antitoxin TakA is an atypical kinase that phosphorylates the TglT toxin, resulting in its neutralization (31). It is noteworthy that *M. tuberculosis* is equipped with distinct eukaryotic-like protein kinases (34, 35), and TakA has very little similarity with these proteins, thus providing the rationale to investigate the role of STPKs (if any) in the regulation of TA modules. During the preparation of this manuscript, Frando et al. reported widespread *O*-phosphorylation of the *M. tuberculosis* H37Rv proteome (44). While their data implicated multiple STPKs in TA modification, this study provides direct biochemical evidence of differential phosphorylation of mycobacterial type II Rel TA proteins via PknK, highlighting a novel underlying regulatory mechanism.

The RelBE toxin-antitoxin system has been extensively studied in *E. coli* where the RelE toxin is activated under the stringent response and specifically cleaves ribosome-associated mRNAs, thereby reducing the global level of translation (51). Likewise, the *M. tuberculosis* RelBE homologs are functional mediators of persistence involved in stress-induced translational control (36) with the toxins exhibiting differential cytotoxic effects (49). While the *M. tuberculosis* RelE toxin is an acutely toxic mRNA interferase, that, in the absence of RelB antitoxin, induces a viable but dormant phenotype in a large proportion of the population (19), the RelK toxin is moderately cytotoxic (18, 49). These inherent differences stem from the structural and sequence divergence of these proteins such that RelBE (RelBE1) and RelFG (RelBE2) are grouped together in the RelBE subfamily within the Rel superfamily, while RelJK (RelBE3) is considered a part of the *E. coli* YefM/YoeB system (49). The resolution of the RelK crystal structure affirmed that the catalytic mechanism of the RelK toxin is closer to the YoeB toxin than the RelE or RelG toxins (49). At present, there is no information regarding the cellular targets or substrates of the RelK toxin. However, a YoeB toxin from *Agrobacterium tumefaciens* was recently shown to cleave both RNA and DNA with similar efficiency (52), suggesting that YoeB-like toxins can act as non-specific, ribosome-independent nucleases. Whether this is true for *M. tuberculosis* RelK toxin remains to be investigated. In accordance with their role in mycobacterial persistence, transcripts specific for *relE* and *relK* toxins and *relF* antitoxin have been detected in *M. tuberculosis*-infected human macrophages (19). Therefore, it is not surprising that these TAs, particularly *relFG* and *relJK*, are significantly upregulated in response to antibiotic exposure (53), nitrogen starvation (36), oxidative stress (36), and in the lungs of infected mice (53) and thus may be subjected to multiple levels of regulatory controls.

A key finding of this study is that PknK, a cytosolic STPK, interacts with RelE and RelJK TA proteins and directs Ser/Thr phosphorylation at multiple sites. We detected three phosphosites on the RelJ antitoxin (Thr21, Ser55, and Ser74) and a single phosphosite (Thr77) on the RelK toxin. In this study, we focused on the phosphorylation of the RelJK module, specifically the RelK toxin. We rationalized that phosphorylation of the RelK toxin could potentially impact its structure, affecting binding with the RelJ antitoxin and/or regulating its catalytic activity. Using MDSs, we analyzed structural changes in the RelK protein as a result of Thr77 modification to understand how phosphorylation changes the movement of protein in different regions. Strikingly, phosphorylation of the Thr77 residue in RelK toxin caused structural perturbation leading to significant changes in its secondary structure evident by the loss of beta sheet and alpha helices. Following up on these observations and additional data from APBS and PCA analysis (Fig. 5), we propose that Thr77 in RelK is key to the structural integrity of RelK, with allosteric communication, electrostatic redistribution, and structural destabilization as probable mechanisms influencing phosphorylation-induced changes in RelK~P structure.

Protein phosphorylation is known to alter protein charge with consequential effects on conformation and functional activity. Expectedly, the presence of a phosphate moiety at the Thr77 residue in RelK disrupts hydrogen bonding and several key interactions with the RelJ antitoxin, as shown in Fig. 5 and 6. Since a greater number of hydrogen bonds between protein-protein complexes correlates with enhanced stability, the significantly reduced hydrogen bond formation potential of RelJK~P (in comparison to non-phosphorylated RelJK complex) is a direct indication of the reduced stability of the phosphorylated protein complex (Fig. 6C). Indeed, protein-protein interaction analysis shows a higher positive energy state for the phosphorylated complex compared to the unphosphorylated complex. The conclusions were verified by a complete loss of binding of RelK~P protein to RelJ antitoxin (Fig. 6D through F). Although the exact role of Thr77 phosphorylation in the catalytic activity of RelK toxin is presently unclear and warrants further investigations, it is evident that PTM of the RelK toxin has consequences on toxin structure and its ability to bind the antitoxin.

Disruption of intermolecular interactions between TA proteins is an obvious way to increase cellular levels of the toxin, which otherwise is neutralized by binding to the antitoxin. During the preparation of this manuscript, Malakar et al. tested this hypothesis and reported that phosphorylation of VapB antitoxin disrupted its interaction with the VapC toxin (54). Our findings resonate with this hypothesis, except that in our case, it is the toxin phosphorylation that interfered with binding to the RelJ antitoxin. In both cases, however, the net outcome is increased cellular level of free toxin, underlining phosphorylation as a mechanism to impede toxin-antitoxin interaction and diminish toxin neutralization. It is likely that the growth defect observed in rescue experiments is a reflection of the number of free toxin molecules arising from incomplete neutralization (Fig. 7B and C). It is pertinent here to note that systematic investigation of the multisite phosphorylation of RelJ is required to comprehensively understand its contribution to the RelJ-RelK interaction dynamics.

With clear benefits to prokaryotes and correspondingly detrimental consequences to their host, a rapid, global TA response could assist in antibiotic evasion and persistence maintenance. Our study sheds light on previously uncharacterized mechanisms related to mycobacterium persistence and has the potential to advance understanding of the current medical enigma of bacterial persistence facilitating the development of new anti-tubercular therapies. We establish Ser/Thr phosphorylation of the toxin component as a means to disrupt TA complex formation, rendering the toxin free. The upregulation of *rel* and *pknK* transcripts under conditions of nitrogen starvation (36, 37) suggests that these cellular environments may be where PknK-mediated phosphorylation of Rel proteins is most physiologically relevant. We predict that other STPKs may similarly act to modify the TA proteins under different activating environments. If this is true, and the cross-interactions between TA systems are extended to other mycobacterial TA modules, then it would result in a highly complex stress-response network, one that will undoubtedly influence *M. tuberculosis* resilience and pathogenic fitness.

## MATERIALS AND METHODS

### Bacterial strains, plasmids, and culture conditions

Table S5 summarizes the strains and plasmids used in this study. For cloning and expression of recombinant proteins, *E. coli* DH5α and *E. coli* BL21 (DE3) strains have been used respectively. *E. coli* was routinely grown in LB broth or LB agar plates at 37°C or in 2× YT medium for large-scale protein purification. *M. smegmatis* was grown in LB broth supplemented with 0.05% Tween 80 and grown at 37°C with aeration at 180 rpm. For the M-PFC assay, *M. smegmatis* transformants were plated on Middlebrook 7H11 agar (Difco) supplemented with 0.5% glycerol, 0.5% glucose, and 0.2% Tween 80 and grown at 37°C. The cultures were supplemented with antibiotics or inducing supplements at the following concentrations: ampicillin (Amp), 100 µg/mL; kanamycin (Kan), 50 µg/mL; hygromycin (Hyg), 150 µg/mL for *E. coli* and 50 µg/mL for *M. smegmatis*; TRIM, 25 µg/mL; isopropyl-β-d-thiogalactopyranoside, 1 mM; glucose, 1%; Ara, 0.2%; ATc, 50 ng/mL and acetamide, 0.2%. All chemicals were procured from Sigma-Aldrich, USA, unless stated otherwise.

### Mycobacterium protein fragment complementation assay

To assess whether Rel toxins or antitoxins interact with PknK in mycobacteria, the M-PFC system was used as described previously (46). Briefly, when two mycobacterial interacting proteins are independently fused with domains of murine dihydrofolate reductase (mDHFR), functional reconstitution of the two mDHFR domains can occur in mycobacteria, allowing for the selection of mycobacterial resistance against TRIM. *M. tuberculosis relB*, *relF*, *or relJ* genes cloned into pUAB100 and pUAB300, episomal mycobacterium-*E. coli* shuttle plasmids, gave rise to fusions of the [F1,2] mDHFR domains to each antitoxin (i.e., RelB_[F1,2]_). *M. tuberculosis relE*, *relG*, or *relK* genes cloned into the integrating mycobacterium-*E. coli* shuttle plasmids pUAB200 and pUAB400 resulted in the fusion of the [F3] mDHFR domain to each toxin (i.e., RelE_[F3]_). Plasmids pUAB100 and pUAB200 generate C-terminal fusions (subscript “-C”), whereas pUAB300 and pUAB400 plasmids generate N-terminal fusions (subscript “-N”). Full-length *pknK* cloned into pUAB300 and pUAB400 resulted in PknK_[F1,2]-N_ and PknK_[F3]-N_.

For all pUAB100 and pUAB200 clones, the GCN4 domains from pUAB100 and pUAB200 were replaced with *rel* DNA sequences. *M. smegmatis* was co-transformed with plasmid constructs that encoded a toxin or antitoxin and *pknK*. As negative controls, a pUAB plasmid containing *relB* or *relE* was co-transformed with an empty vector (i.e., RelB_[F1,2]-C_/pUAB200), whereas a positive control was provided by the GCN4_[F1,2]_/GCN4_[F3]_ (GCN4 leucine zipper, *Saccharomyces cerevisiae* interacting domains in pUAB100 and pUAB200) (55). All transformants were plated on 7H11-Kan-Hyg plates and incubated at 37°C for 3 days. Transformants were re-streaked onto 7H11-Kan-Hyg and 7H11-Kan-Hyg-TRIM in duplicate and incubated at 37°C for 3–5 days.

### Expression and purification of Rel TA and PknK proteins

The wild-type and mutant *M. tuberculosis* PknK and Rel TA proteins were purified as recombinant His-tagged proteins by Ni^2+^-NTA affinity chromatography. While Rel antitoxin proteins were purified under native conditions, the wild-type (RelE, RelG, and RelK) and mutant RelK_Mut_T77A toxins formed inclusion bodies and, thus, were purified under denaturing conditions followed by on-column refolding. Recombinant proteins were purified using standard methods described in the supporting information.

### *In vitro* kinase assay

*In vitro* kinase assay was performed essentially as described previously (56) except that phosphoprotein staining was used to visualize phosphorylation. Briefly, purified wild-type PknK protein kinase (1 µM) and substrate proteins (30 µM) were mixed in a tube containing 1× kinase buffer (25 mM HEPES pH 7.4, 15 mM MgCl_2_, 5 mM MnCl_2_, 1 mM DTT, and 1 mM ATP) and incubated at 30°C for 30 min. The reaction was stopped using 5× SDS sample loading dye, followed by SDS PAGE for separation of proteins. After electrophoresis, the gel was processed for staining by ProQ Diamond Phosphoprotein Gel Stain (Thermo Fisher Scientific) as per the manufacturer’s protocol. The ProQ-stained gels were scanned with the Typhoon Trio Variable Mode imager (GE Healthcare). The gel was then stained with CBB to visualize the protein bands. The kinase assays were done in triplicate, and densitometric analysis was done using the ImageJ software to determine relative phosphorylation. The substrate concentration from CBB-stained gels was normalized across all gels before the calculation of fold change in signal intensity.

### Mass spectrometry

For the identification of phosphorylated residues, *in vitro* kinase assays were performed as described above, and the proteins were resolved by SDS-PAGE. Individual proteins alone were processed similarly as negative controls. The gel bands observed after CBB staining were excised and subjected to LC-MS/MS analysis. Mass spectrometry was performed using standard procedures and protocols described in the supporting information.

### Site-directed mutagenesis

To create a phosphodeficient mutant of RelK, threonine at position 77 was replaced with alanine using the primer pair: forward primer 5′[P]-CGACGAAGTC**G**CGATGCTGAAG-3′ and reverse primer; 5′[P]-TCGCCCGCTCGATACACC-3′. To create a phosphomimetic mutant of RelK, threonine at position 77 was replaced with glutamate using the primer pair: forward primer 5′-GACGAAGTC**GA**GATGCTGAAGGCCCG-3′ and reverse primer; 5′-CGGGCCTTCAGCATC**TC**GACTTCGTC-3′. Site-directed mutagenesis (SDM) was performed with the Phusion SDM Kit (Thermo Fisher Scientific) as per the manufacturer’s instructions to generate the above-specified mutations in expression vectors as listed in Table S5. The plasmids containing the desired mutation were confirmed by DNA sequencing.

### All-atomistic molecular dynamics simulations of the RelJK TA module

All-atomistic molecular dynamic simulation was performed for the *M. tuberculosis* RelJK TA system (PDB:3OEI) comprising RelK (Toxin) and RelJ (Antitoxin) proteins (49). MDS was performed for the following *M. tuberculosis* proteins: (i) RelK control (unphosphorylated), (ii) RelK~P (phosphorylated at Thr77), (iii) RelJ control (unphosphorylated), (iv) RelJK complex (unphosphorylated control), and (v) RelJ-RelK~P complex (RelK phosphorylated at Thr77) with GROMACS (GROningen MAchine for Chemical simulations) software package (57, 58). Phosphorylation of RelK toxin at Thr77 was introduced using the pdb manipulator tool of CHARMM GUI (59, 60). Details of the parameters and tools used for MDSs are described in the supporting information.

The trajectories obtained from the MDSs were analyzed to explore the structural parameters, including RMSD, RMSF, and Rg. The plots were generated using Python code. Number of hydrogen bonds was detected by analyzing the trajectories with the program *gmx hbond* of the GROMACS software between the RelK chain and RelJ chain for the control and the phosphorylated complex.

The residue interaction network was constructed using the final structure obtained after the MDS. A Python script was used to identify interactions between residues based on predefined bond cutoff distances, categorizing the interaction types accordingly. The interaction data were then formatted into a .sif (Simple Interaction Format) file, which was imported into Cytoscape for visualization and network analysis. Electrostatic potential maps were generated using the APBS (61) PDB2PQR tools to assess the charge distribution changes upon phosphorylation. The three-dimensional structures of the unphosphorylated and phosphorylated protein states were obtained after the molecular dynamics simulations. PCA was performed using ProDy (62–64) and visualized in the Visual Molecular Dynamics (VMD) (65) Normal Mode Wizard to extract dominant modes of motion from the molecular dynamics trajectory. The atomic fluctuation covariance matrix was computed from the aligned trajectory, followed by eigenvalue decomposition to identify principal components representing large-scale conformational changes.

### *In vitro* binding studies

Purified proteins were incubated in combinations of RelJ and RelK variants (RelK_WT_, RelK_Mut_T77A, or RelK~P) in the equimolar ratio of 1:1 unless stated otherwise. Protein-protein interaction was allowed for 30 min at 30°C in 1× binding buffer (50 mM Tris pH 7.4, 150 mM NaCl, and 10 mM MgCl_2_) as suggested previously (66) except for the addition of Tween 20. For obtaining RelK~P, the *in vitro* kinase reaction with PknK was further subjected to purification using a molecular weight cut off to remove the kinase. RelK~P purified in this manner was dialyzed to remove salts and excess ATP. Reaction mixtures were added with 5× native loading dye and separated on an 8% Native PAGE in two replicate experiments. Gels were stained using a CBB staining solution.

### Isothermal titration calorimetry

ITC measurements were performed using a Microcal PEAQ-ITC (Malvern Panalytical) instrument as described (67). The RelJ protein suspended in 10 mM Tris, pH 7.4 (3 μM) was kept in the sample cell, and RelK~P (60 µM) in the same buffer was placed in a syringe of volume 40 µL. RelK~P was added sequentially in 2 µL aliquots (for a total of 18 injections, 4 s duration) into the cell with 180 s of spacing time. The experiment was performed at 25°C. The injections were done from a stirring syringe rotating at 750 rpm, with detection on high feedback. The heat of dilution was determined in independent experiments by titrating a RelK~P solution into the same buffer in the cell. The generated heat burst curve (microwatts per second) with respect to time was plotted to give total heat per injection. The data were fit according to adjusted baseline parameters using MicroCal PEAQ ITC Analysis software.

### Phenotypic growth and rescue studies

*E. coli* BL21 (DE3) cells were co-transformed with the expression vectors, pLTA(*relJ*)20 and pCAK(*relK* or *relK*T77A or *rel*KT77E)10 expressing RelJK_WT_ or RelJK_Mut_T77A or RelJK_Mut_T77E protein pairs and grown overnight in 50 mL tubes containing LB media supplemented with Amp and Kan at 37°C with shaking at 180 rpm as described previously (18). Secondary inoculation was done with 1% of primary inoculum and grown till OD_600 nm_ ~ 0.2. The cultures were split, and one part was supplemented with Ara to induce toxin production, or with ATc and Ara to induce co-expression of the antitoxin and toxin proteins as described previously (18). Growth was followed by measuring OD_600 nm_ every 60 min for 6 h, and CFU analysis was done at the end timepoint on LB agar plates containing relevant antibiotics.

For rescue studies in mycobacteria, the *M. tuberculosis relJK* ORFs were cloned under the acetamide-inducible promoter in the hygromycin-resistant, *E. coli*-mycobacterial site-specific integrative shuttle vector, pJFR19 (68). The recombinant plasmid pJFR19::*relJK*_WT_ was subjected to site-directed mutagenesis to yield recombinant vectors carrying *relJK*_Mut_T77A and *relJK*_Mut_T77E gene pairs followed by electroporation in *M. smegmatis*. The transformed cells were cultivated in LB media supplemented with Hyg and Tween 80 at 37°C with aeration and grown till OD_600 nm_ ~ 0.3, after which they were split. One part was induced with 0.2% acetamide, allowing induction of the *relJK* or *relJK*_Mut_T77A or *relJK*_Mut_T77E genes. Viable counts were determined by drop plating 10-fold serial dilutions on LB Agar plates supplemented with Hyg at 6 h, 18 h, and 24 h time points. For a particular strain at each time point, viable counts obtained under inducing conditions were normalized to the counts observed in uninduced cells grown in parallel. Data are represented as mean ± SD from two independent biological replicates each having two technical replicates.

### Statistical analysis

Statistical analyses were performed using Student’s *t*-test for growth studies and for ProQ-phosphostaining reactions and by Mann-Whitney *U* test for differences in the medians for hydrogen bond formation observed in RelJK complexes (control vs phosphorylated). GraphPad Prism 9.0 was used to plot the growth data and calculate statistical significance. A *P* value of <0.05 was considered statistically significant.

## Data Availability

The data used to support the findings of this study are included within the article or provided in the supplemental material. Any further details required will be provided upon request to the corresponding author.

## AFTER EPUB

*[This article was published on 17 June 2025 with an error in the Invited Editor affiliation. The affiliation was corrected in the current version, posted on 23 June 2025.]*

## SUPPLEMENTAL MATERIAL

Video File legends mbio.01068-25-s0001.pdf Legends for the supplemental videos.

Supplemental Material mbio.01068-25-s0002.pdf Supplemental figures, tables, and methods.

Video S1 mbio.01068-25-s0003.mp4 RelK PCA animation.

Video S2 mbio.01068-25-s0004.mp4 RelK-P PCA animation with Thr 77 marked.

ASM does not own the copyrights to Supplemental Material that may be linked to, or accessed through, an article. The authors have granted ASM a non-exclusive, world-wide license to publish the Supplemental Material files. Please contact the corresponding author directly for reuse.
