# Supplementary material for: Ser/Thr phosphorylation of Mycobacterium tuberculosis type II RelK toxin by PknK destabilizes TA interaction and interferes with toxin neutralization
Source: mBio. 2025 Jun 17;16(7):e01068-25. doi: 10.1128/mbio.01068-25 (PMC12239584; doi:10.1128/mbio.01068-25)
Supplement: Video File legends — Legends for the supplemental videos. [file mbio.01068-25-s0001.pdf]

Supplemental Data

Figure S4 (A) Video S1:
Principal Component Analysis (PCA) of RelK (unphosphorylated). The red ribbon represents the protein backbone, with green arrows indicating principal motion vectors derived from PCA. The mobility color scale (0–1) represents normalized mobility scores, with higher values indicating greater structural flexibility. In RelK, the highest mobility is observed in residues **60–67** and **83–85.** Thr77 (highlighted as a yellow sphere) marks a key site of interest.

Figure S4 (B) Video S2:
Principal Component Analysis (PCA) of RelK~P (Phosphorylated). The red ribbon represents the protein backbone, with green arrows indicating principal motion vectors derived from PCA. The mobility color scale (0–1) represents normalized mobility scores, with higher values indicating greater structural flexibility. In RelK~P, highest mobility is most pronounced in residues **51–57**. Thr77 (highlighted as a yellow sphere) marks a key site of interest.
