# Supplementary material for: Ser/Thr phosphorylation of Mycobacterium tuberculosis type II RelK toxin by PknK destabilizes TA interaction and interferes with toxin neutralization
Source: mBio. 2025 Jun 17;16(7):e01068-25. doi: 10.1128/mbio.01068-25 (PMC12239584; doi:10.1128/mbio.01068-25)
Supplement: Supplemental Material — Supplemental figures, tables, and methods. [file mbio.01068-25-s0002.pdf]

Ser/Thr phosphorylation of *Mycobacterium tuberculosis* Type II RelK toxin by PknK destabilizes TA interaction and interferes with toxin neutralization

List of Supporting Information:

- Figures S1- S7
- Tables S1-S5
- Supporting Experimental Procedures
- References

Figure S1

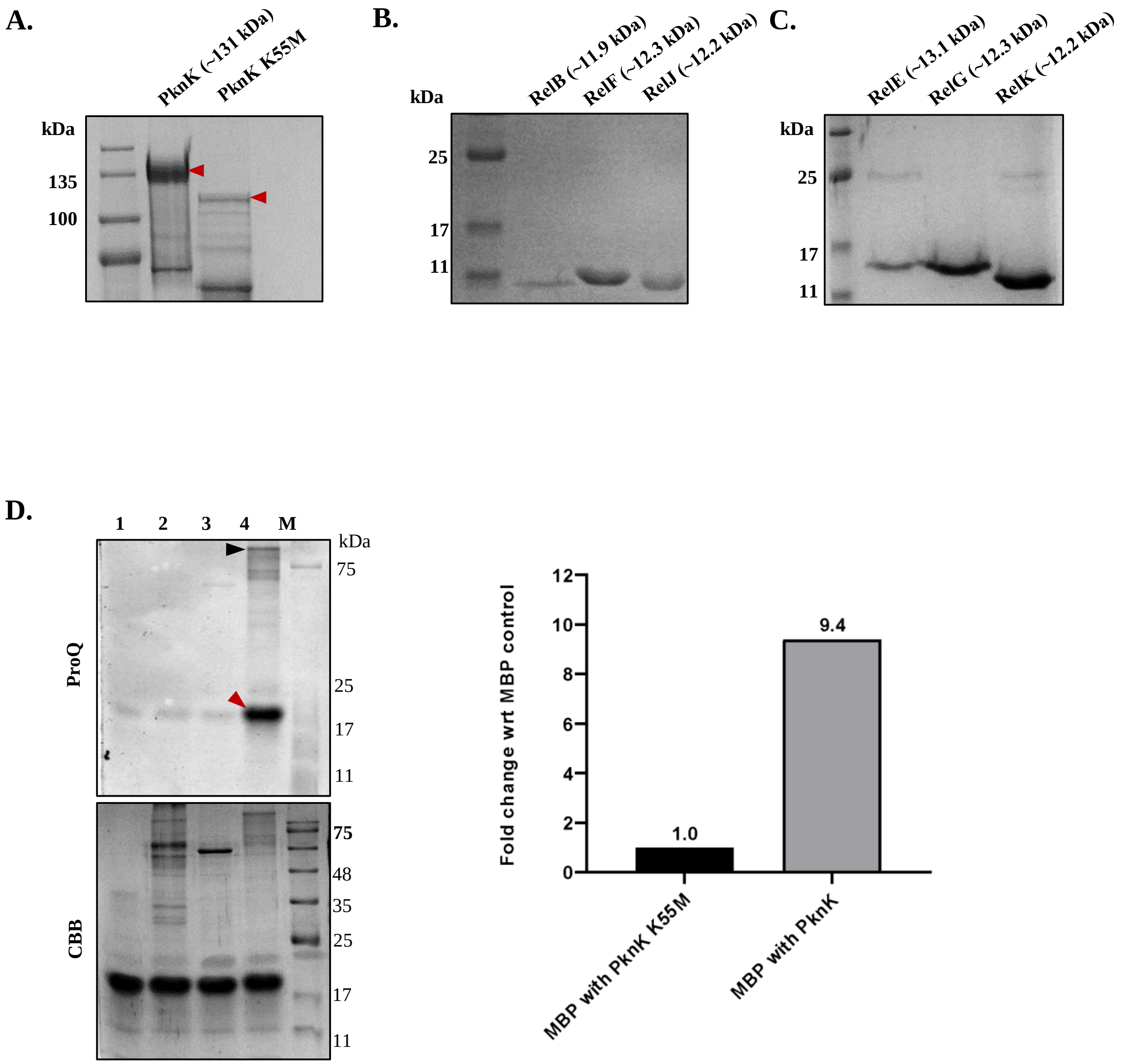

Figure S1.

Purification of **(A)** Full length PknK and PknK K55M Mutant **(B)** Antitoxins (RelB, RelF, RelJ) and **(C)** Toxins (RelE, RelG, RelK) **(D)** *In vitro* kinase assays of PknK Kinase Domain (PknK KD), PknK K55M Mutant and PknK with Myelin Basic Protein (MBP) have been performed. Lane 1 shows MBP alone, Lanes 2-4 show MBP with PknK K55M, PknK KD and PknK respectively. Red arrow on the top gel panel shows transphosphorylation of MBP by PknK. Black arrow shows PknK. (12% Gel stained with ProQ^{TM} Diamond on the top and CBB on the bottom). Densitometry analysis of the gel revealed a ~9.4-fold increase in the intensity of phosphosignal on MBP post transphosphorylation by wild type PknK.

Figure S2

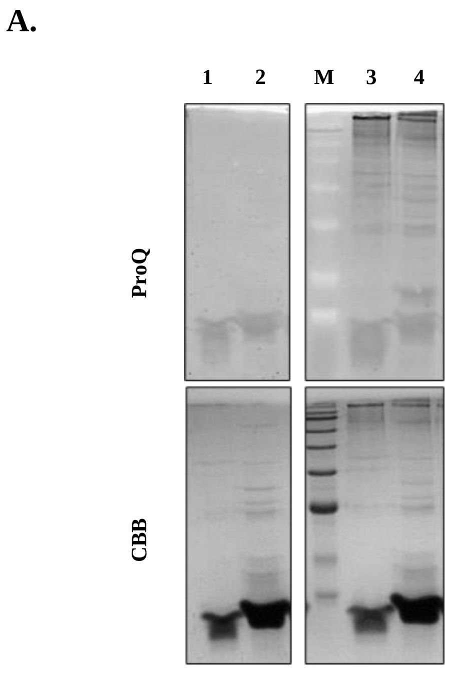

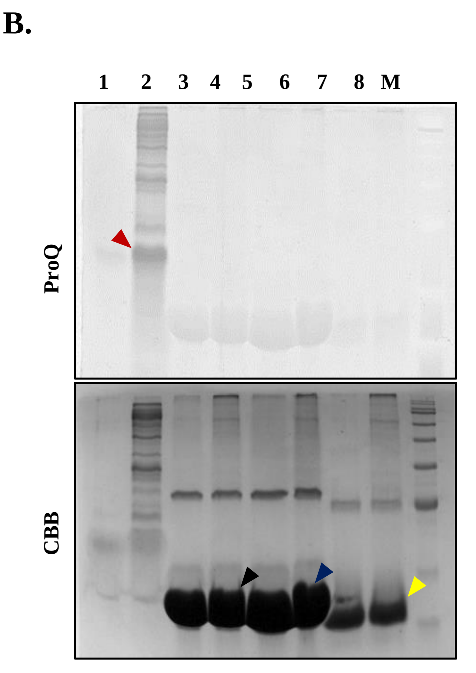

Figure S2.

A. ***In vitro*** **kinase assay of RelB and RelF with PknK**. RelB and RelF alone (Lanes 1-2), and with PknK (Lanes 3-4). No change in signal intensity was observed with RelB and RelF antitoxins in presence of PknK.
B. ***In vitro*** **kinase assay of RelE, RelK & RelJ with PknK K55M Mutant.** Lanes 1 and 2 are MBP (positive control) without and with wild type PknK, respectively. Lanes 3, 5 and 7 are RelE, RelK and RelJ respectively without phosphorylation deficient PknK K55M mutant protein while lanes 4, 6 and 8 are with the mutant PknK kinase. Red arrow indicates positive control, Black, Blue and Yellow arrows show RelE, RelK and RelJ on CBB stained gel, respectively. No signal was observed for RelE, RelJ and RelK on ProQ-stained gels with the mutant PknK kinase.

Figure S3

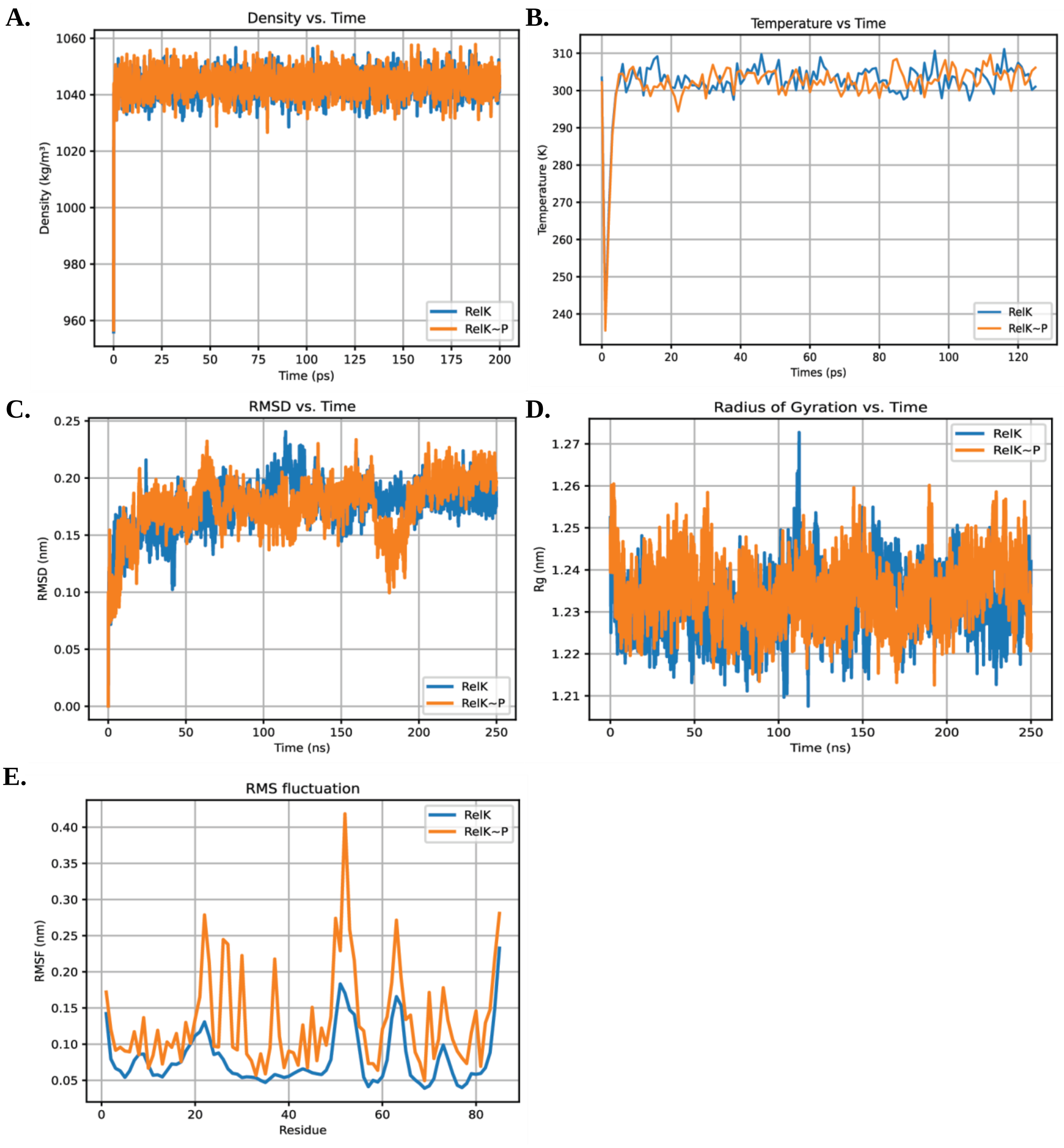

Figure S3.

(A) Density of solvated RelK (blue) and RelK~P (orange) across time during equilibration. **(B)** Temperature of solvated RelK (blue) and RelK~P (orange) across time during equilibration. **(C)** Root mean square deviation (RMSD) of the backbone atoms of RelK (blue) and RelK~P (orange). **(D)** Radius of gyration of RelK (blue) and RelK~P(orange). **(E)** Root mean square fluctuation (RMSF) of RelK (blue) and RelK~P(orange).

Figure S4

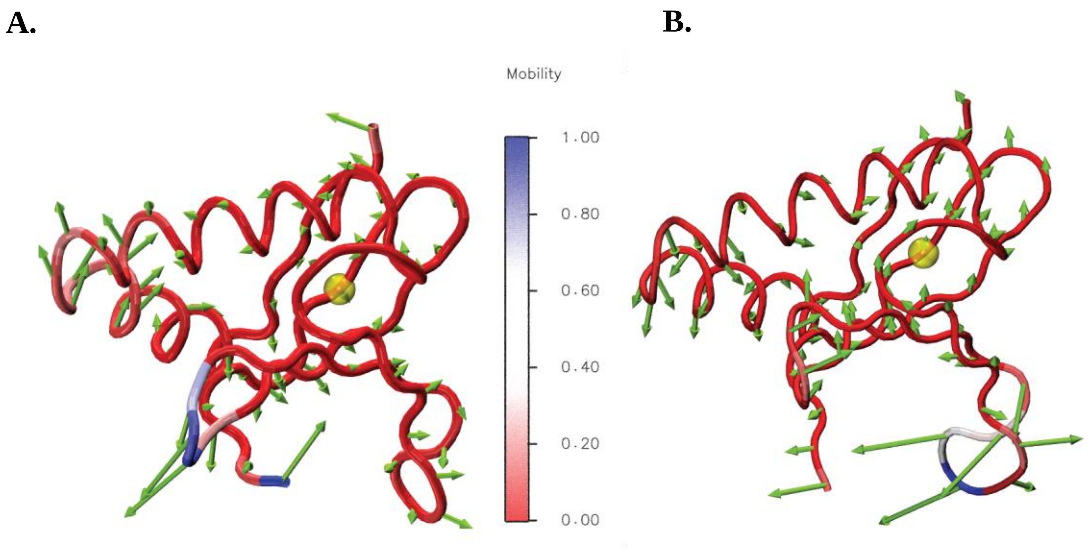

Figure S4.

Principal Component Analysis (PCA) of (A) RelK (unphosphorylated) and **(B)** RelK~P (phosphorylated).The red ribbon represents the protein backbone, with green arrows indicating principal motion vectors derived from PCA. The mobility color scale (0–1) represents normalized mobility scores, with higher values indicating greater structural flexibility. In RelK, the highest mobility is observed in residues **60–67** and **83–85**, while in RelK~P, it is most pronounced in residues **51–57**. Thr77 (highlighted as a yellow sphere) marks a key site of interest. Phosphorylation alters dominant motion patterns, suggesting structural rearrangements that may impact protein dynamics and functional interactions. Click on the underlined embedded links shown above to access the animation movie or see .mp4 video files **Figure S4 (A) Video S1** and **Figure S4 (B) Video S2** for RelK unphosphorylated and RelK~P, respectively.

Figure S5

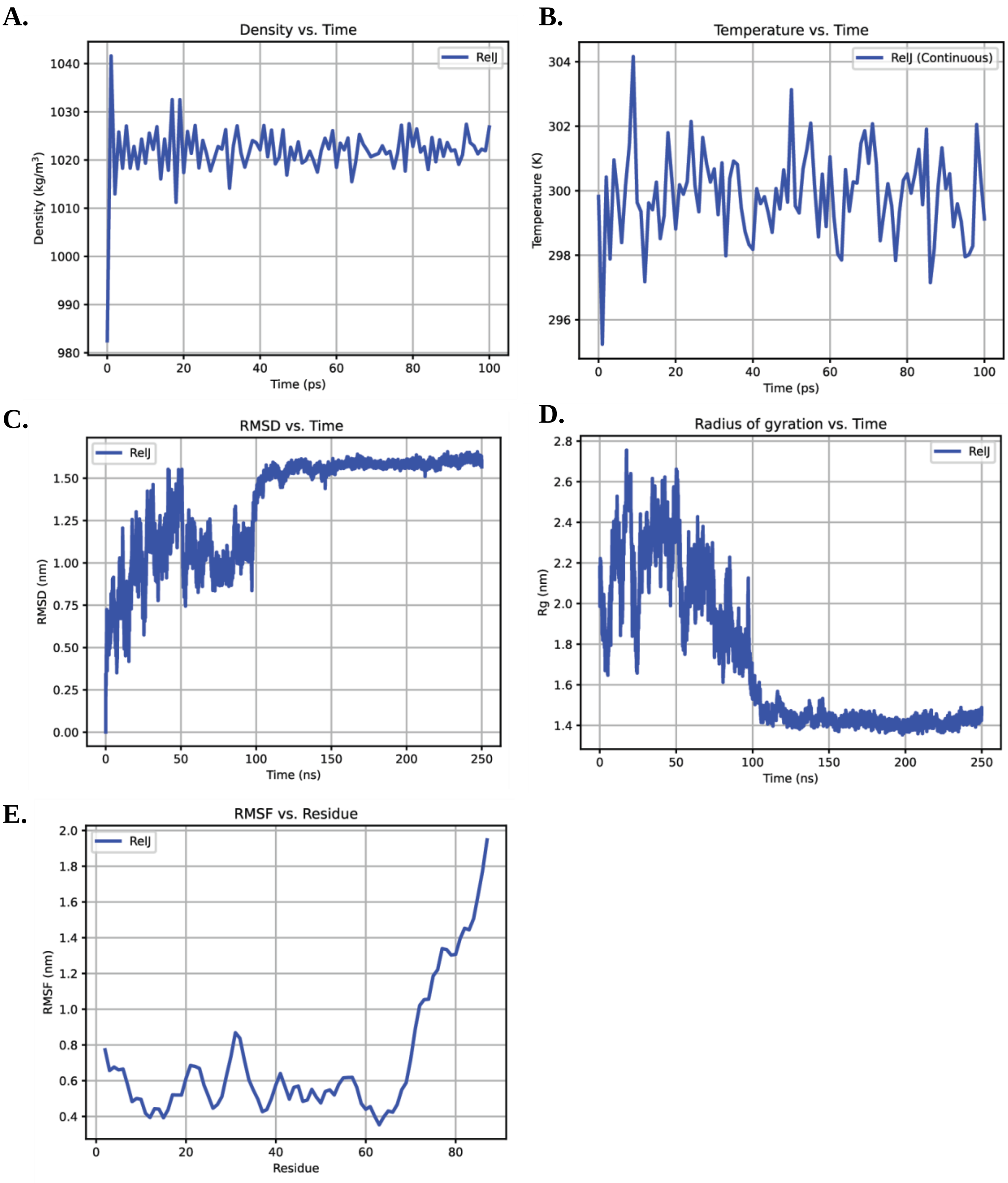

Figure S5.

(A) Density of solvated RelJ across time during equilibration. **(B)** Temperature of solvated RelJ across time during equilibration. **(C)** Root mean square deviation (RMSD) of the backbone atoms of RelJ. **(D)** Radius of gyration of RelJ. **(E)** Root mean square fluctuation (RMSF) of RelJ.

Figure S6

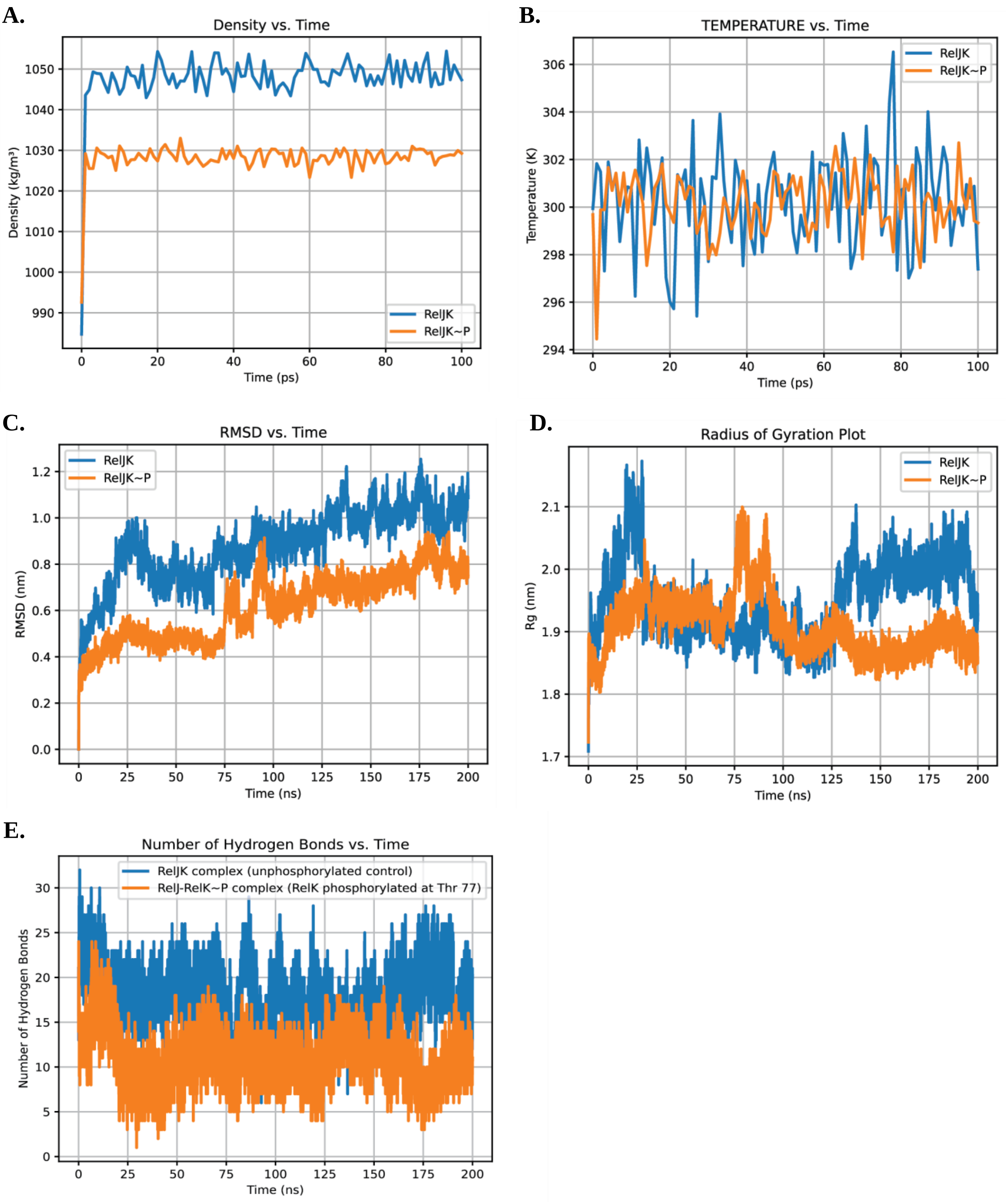

Figure S6.

(A) Density of solvated RelJK (blue) and RelJK~P (orange) across time during equilibration. **(B)** Temperature of solvated RelJK (blue) and RelJK~P (orange) across time during equilibration. **(C)** Root mean square deviation (RMSD) of the backbone atoms of RelJK (blue) and RelJK~P (orange). **(D)** Radius of gyration of RelJK (blue) and RelJK~P(orange). **(E)** Plot of hydrogen bond formation during the MD simulation period between RelJ and RelK in the RelJK (unphosphorylated control) shown in blue and RelJ-RelK~P (RelK phosphorylated at Thr77) shown in orange**.**

Figure S7

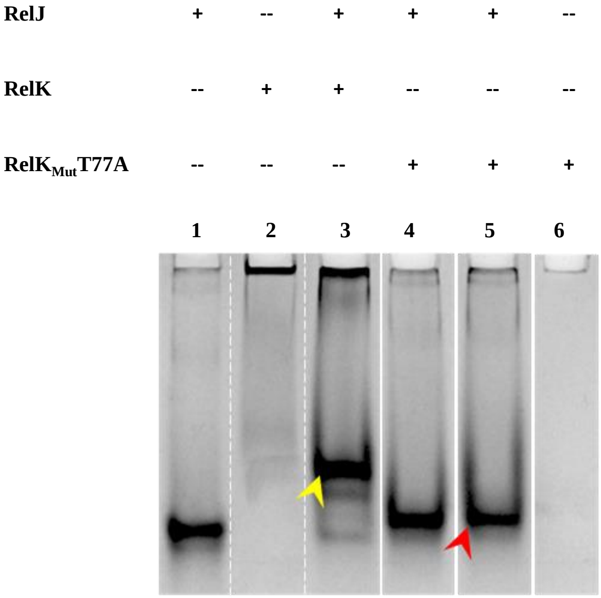

Figure S7.

Effect of phosphorylation on interaction of wild type and mutant RelK with RelJ antitoxin protein on 8 % Native PAGE.

Binding of RelJ with RelK$_{WT}$ or RelK$_{Mut}$T77A proteins. Lanes 1, 2 and 6 are RelJ, and RelK$_{WT}$ and RelK$_{Mut}$T77A alone, respectively. RelJ was incubated with RelK$_{WT}$ in equimolar ratio (Lane 3) and with RelK$_{Mut}$T77A in increasing molar ratios (Lane 4 and 5). Yellow arrow indicates the shift observed upon binding of RelJ with RelK wild type proteins. On the contrary, only a slight shift was observed when RelJ was incubated with RelK$_{Mut}$T77A (red arrow).

Table S1. Compilation and comparison of predicted Ser/Thr/Tyr phosphorylation sites in *M. tuberculosis* Rel TA proteins.

Protein	Antitoxin/Toxin	[a]NetPhosBac 1.0	[b]MusiteDeep	[c](1)
**RelB**	Antitoxin	**14S**<br>30T	**14S**<br>57T<br>**78S**	**78S**
**RelE**	Toxin	**58S**<br>86S<br>**88S**	**2S**<br>7Y<br>**58S**<br>**88S**	**2S**
**RelF**	Antitoxin	**None**	63S<br>73S	**None**
**RelG**	Toxin	10T<br>35S<br>**54S**<br>72T	**54S**	None
**RelJ**	Antitoxin	74S*	2S<br>4S<br>43Y<br>55S*<br>79S	21T*
**RelK**	Toxin	3S<br>**54S**	77T*	**54S**

[a] *S/T phosphorylation sites as predicted by NetPhosBac1.0*

(https://services.healthtech.dtu.dk/services/NetPhosBac-1.0/)

[b] *S/T/Y phosphorylation sites as predicted by MusiteDeep (https://www.musite.net)*

[c] *Data compiled from literature* (1)

Common phosphosites are highlighted in Bold.

*Sites identified in this study are marked with an asterisk**

Table S2. Identification of phosphosite in RelK by LCMS/MS

Master	Accession	Description	Sequence	Coverage [%]	# Peptides	# PSMs	# Unique Peptides	# AAs	MW [kDa]	calc. pI	Score MS Amanda 2.0: MS Amanda 2.0	Score Sequest HT: Sequest HT	# Peptides (by Search Engine): MS Amanda 2.0	# Peptides (by Search Engine): Sequest HT	Biological Process	Cellular Component	Molecular Function	Pfam IDs	Entrez Gene ID	Gene Symbol	Gene ID	# Protein Pathway Groups	Found in Sample: [S2] F2: Sample	# Protein Groups	Modifications
**Master Protein**	**P9WF09**	**culosis (strain ATCC 25618 / I**	**GEIQRDPFSGIGKPEPLQGEL**	**96**	**15**	**50**	**15**	**85**	**10.1**	**6.55**	**4394.14**	**71.76**	**10**	**9**	**otion;other metabolic process**		**ucleic acid binding activity;otl**	**Pf06769**	**45427357; 888139**	**relK**	**mtu:Rv3358; O50387; P64528**	**0**	**High**	**1**	**Phospho [T77(100)]**
Master Protein	**P9WGL1**	OS=Mycobacterium tuberculosis	RLVTDAGHELRTPLTSLRTNVEL	4	1	2	1	504	54.4	9.67		7.74		1	sm;other metabolic processes;si		ase activity;other molecular func	Pf00512, Pf00672, Pf02518	885062	mprB	mtu:Rv0982; O53895; P9WGL1	0	High	1	
Master Protein	**P9WIG5**	erculosis (strain ATCC 25618 / I	NGGESGGTGGVGGAGGNAVL	3	1	1	1	914	74.3	5		3.96		1				Pf00934	885325	wag22	mtu:Rv1759c; O06794; P0A686	0	High	1	
Master Protein	**P9WP25**	acterium tuberculosis (strain AT	PMLLYDIPGRSAVPIEPDTIRAL	8	1	1	1	300	30.8	5.8		3.95		1	other metabolic processes	cytosol	other molecular function	Pf00701	45426740; 888289	dapA	mtu:Rv2753c; O33295; P63945	0	High	1	
Master Protein	**P9WN33**	Mycobacterium tuberculosis (st	SEIDVAVASAEAIGARHIAKVVS	5	1	1	1	652	72.1	6.79		2.9		1	other metabolic processes		other molecular function	Pf00733, Pf13537	888472	asnB	mtu:Rv2201; P64247; P9WN33	0	High	1	Phospho [T166(100); T171(100); S175(100)]
Master Protein	**P9WF11**	ycobacterium tuberculosis (stra	YTQGVTGVLAGSSTYPGAAVLC	4	1	1	1	473	46.8	6.05		2.12		1	other metabolic processes		other molecular function	Pf01256, Pf03853	887571	nnr	L5; mtu:Rv3433c; O06250; P9\	0	High	1	
Master Protein	**I6YCF3**	tuberculosis (strain ATCC 2561	DVDTPLEVTAVRADGWLGDLL	1	1	8	1	1013	111.5	5.9	1318.32		1		cell organization and biogenesis		id binding activity;other molecu	Pf00176, Pf00271, Pf12419	888635	helZ	I6YCF3; L0TBI9; mtu:Rv2101	0	High	1	
Master Protein	**P71552**	um tuberculosis (strain ATCC 25	LVVASANPEDYTNRGRIITPIKD	1	1	1	1	459	49.6	5.38	210.68		1		other biological processes		ase activity;other molecular func	Pf00158	885405		A76; L0T5E7; mtu:Rv0958; P71	0	High	1	

Table S3. Identification of phosphosite in RelJ by LCMS/MS

Accession	Description	Sequence	Exp. q-value: MS Amanda 2.0	Exp. q-value: Sequest HT	Coverage [%]	# Peptides	# PSMs	# Unique Peptides	# AAs	MW [kDa]	calc. pI	Score MS Amanda 2.0: MS Amanda 2.0	Score Sequest HT: Sequest HT	# Peptides (by Search Engine): MS Amanda 2.0	# Peptides (by Search Engine): Sequest HT	Biological Process	Cellular Component	Molecular Function	Pfam IDs	Entrez Gene ID	Gene Symbol	Gene ID	# Protein Pathway Groups	Found in Sample: [S11] F11: Sample	# Protein Groups	Modifications
P9WLU7	Uncharacterized protein Rv1546 OS=Mycoba	MASVELSADVPISPQDTWDHVS	0.167	0.167	8	1	4	1	143	15.3	6.8	219.47	0	1	1				Pf10604	886394		546; P64873; F	0	High	1	
P9WFF7	Decaprenyl diphosphate synthase OS=Mycob	MARDARKRTSSNFPQLPPAPDD	0	0.2	6	1	4	1	296	33.8	9.23	302.77	1.73	1	1	other metabolic processes	membrane;other membrane	her molecular functi	Pf01255	888964	uppS	361c; O05837;	0	High	1	Phospho [S169(100)]
P9WM39	Esterase Rv1288 OS=Mycobacterium tubercu	MVSTHAVVAGETLSALALRFYGD	0	0.156	2	1	6	1	456	49.6	5.69	318.04	0	1	1	other metabolic processes	non-structural extracellular	her molecular functi	0756, Pf01	886974		:Rv1288; P9WI	0	High	1	
P9WJQ5	Molybdopterin molybdenumtransferase 2 OS	MRSVQEHQRVVAEMMRACRPI		0.222	6	1	2	1	405	42	5.96		1.8		1	metabolism;other metabolic pro	cytosol;other cell componen	her molecular functi	, Pf03453,	886348	moaE2	:Rv0438c; O537	0	High	1	Phospho [T221(98.7)]
P9WQE7	Phenolphthiocerol/phthiocerol polyketide synt	MTGSISGEADLRHWLIDYLVTNIC	0.235		0	1	1	1	1876	198.7	5.36	181.3		1		tion and biogenesis;other metabo	other cell component	her molecular functi	, Pf02801,	888183	ppsA	:Rv2931; P9W	0	High	1	
P71815	histidine kinase OS=Mycobacterium tubercul	MARHLRGRLPLRVRLVAATLILV	0.25	0.152	2	1	6	1	485	52	7.08	175.99	0	1	1	sm;other metabolic processes;si	ma membrane;other membr	kinase activity	, Pf00672,	888775	phoR	2; mtu:Rv0758;	0	High	1	
P95217	Possible transcriptional regulatory protein OS	MAQAHSAPLTGYRIAVTSARRAE		0.125	3	1	1	1	381	40.7	7.11		0		1	cesses;signal transduction;other		ding activity;other m	0486, Pf02	886651		; mtu:Rv0260c;	0	High	1	Phospho [T364(100)]
O53203	NAD-specific glutamate dehydrogenase OS=	MTIDPGAKQDVEAWTTFTASADI		0.119	2	1	1	1	1624	176.8	5.83		0		1	other metabolic processes		her molecular functi	Pf05088	887437	gdh	; mtu:Rv2476c;	0	High	1	
P9WPF1	Alpha-pyrone synthesis polyketide synthase-l	MNVSAESGAPRRAGQRHEVGL	0		4	1	2	1	393	42	5.87	241.4		1		other metabolic processes		her molecular functi	0195, Pf02	886797	pks18	372; P9WPF1;	0	High	1	Phospho [S7(98.9)]
P9WG81	Bifunctional protein FolD OS=Mycobacterium	MGAIMLDGKATRDEIFGDLKQRV		0.122	7	1	1	1	281	29.5	6.44		0		1	other metabolic processes	cytosol	her molecular functi	0763, Pf02	7355; 88	folD	356c; O50385;	0	High	1	Acetyl [N-Term]
P9WL21	Uncharacterized protein Rv2917 OS=Mycoba	MLHFTAATSRFRLGRERANSVR	0	0.116	2	1	3	1	602	65.8	9.25	233.42	0	1	1			ding activity;other m	Pf04851	887758		2917; P9WL21;	0	High	1	
I6Y4V2	Abortive phage infection protein OS=Mycoba	MSRLRALSLAAGLVGWSLVSPR	0.1	0.125	6	1	2	1	209	21.6	12.1	224.21	2.51	1	1	metabolism;other metabolic pro	other membranes	her molecular functi	Pf02517	885330		Y4V2; mtu:Rv08	0	High	1	
P96214	FHA domain-containing protein OS=Mycobac	MAGERKVCPPSRLVPANKGSTC	0.227		5	1	3	1	392	41.1	8.21	167.42		1						886197		); mtu:Rv3863;	0	High	1	Phospho [T209(100); T223(100)]
P9WGU7	Probable monoacyl phosphatidylinositol tetra	MGVPSPVRRVCVTVGALVALAC		0.179	3	1	3	1	635	66.1	5.64		0		1	ther metabolic processes;transpo	other cell component	transporter activity	Pf00496	5138; 88	lpqW	166; O50422; P	0	High	1	Phospho [Y164(100)]
P9WI65	Serine/threonine-protein kinase PknK OS=My	MTDVDPHATRRDLVPNIPAELLE	0	0	46	71	526	71	1110	119.3	5.81	22663.68	190.93	60	64	other metabolic processes;other	acellular;plasma membrane	ctivity;other molecula	0069, Pf13	888659	pknK	:Rv3080c; P95	0	High	1	Phospho [Y41(100); T59(100); T87(99.3); T113(100); T145(100); T240(100); T274(100); T569(100); T623(100); T733(99.3); T768(100); T779(100); T829(100)]
P9WJF1	Decaprenylphosphoryl-beta-D-ribose oxidase	MLSVGATTTATRLTGWGRTAPS	0.385		3	1	1	1	461	50.1	7.65	141.9		1		enesis;other metabolic processes	other cell component	her molecular functi	1565, Pf04	886125	dprE1	790; P72056; F	0	High	1	Phospho [T416(99.5)]
I6Y498	Probable anion transporter ATPase OS=Myco	MSVTPKTLDMGAILADTSNRVVV	0	0	2	1	9	1	386	41.4	6.09	337.84	3.54	1	1			her molecular functi	Pf02374	885317		Y498; mtu:Rv36	0	High	1	
P9WF11	Bifunctional NAD(P)H-hydrate repair enzyme	MRHYYSVDTIRAAEAPLLASLPD		0.182	4	1	2	1	473	46.8	6.05		1.71		1	other metabolic processes		her molecular functi	1256, Pf03	887571	nnr	:Rv3433c; O062	0	High	1	Phospho [S20(100)]
P9WH51	30S ribosomal protein S17 OS=Mycobacteriu	MAEAKTGAKAAPRVAKAAKAAP	0.214	0.114	8	1	2	1	135	14.7	10.6	209.01	0	1	1	metabolism;other metabolic pro	ytosol;translational apparatu	ding activity;other m	Pf00366	888391	rpsQ	:Rv0710; P950	0	High	1	
O53645	Multidrug efflux ATP-binding/permease protei	MRTNCWWRLSGYVMRHRRDLL		0.109	2	1	1	1	1194	129.2	9.13		0		1	ansport;other biological processe		activity;other molecu	0005, Pf00	886790		:Rv0194; mtv:F	0	High	1	Phospho [T971(99.1)]
O53332	Putative toxin HigB3 OS=Mycobacterium tube	MAVILLPQVERWFFALNRDAMA		0	36	1	1	1	114	12.7	5.91		3.76		1				Pf05973	888795	higB3	3; mtu:Rv3182;	0	High	1	Phospho [T37(98.4)]
L0TAD5	Probable O-methyltransferase Rv1703c OS=I	MAAGIRNITTTGQIGDGREAAAVI	0.267	0.106	5	1	2	1	233	25.2	5.6	185.61	0	1	1	other metabolic processes		her molecular functi	Pf01596			L0TAD5	0	High	1	
O05598	Putative ligase Rv1013 OS=Mycobacterium t	MSRFTEKMFHNARTATTGMVTG		0.091	7	1	2	1	544	58.4	5.3		2.22		1	other metabolic processes		her molecular functi	0501, Pf14	886035	pks16	; mtu:Rv1013;	0	High	1	
O33263	Probable oxidoreductase OS=Mycobacterium	MTSLQGKVVFITGAARGIGAEVA	0.217	0.214	3	1	5	1	293	31.3	9.51	165.98	1.86	1	1			her molecular functi	Pf00106	6104; 88		8Q7; mtu:Rv21	0	High	1	
P9WHJ3	Protein RecA OS=Mycobacterium tuberculos	MTQTPDREKALELAVAQIEKSYG	0.25	0.211	2	1	8	1	790	85.3	6.43	183.63	1.79	1	1	;other metabolic processes;stres		ding activity;other m	, Pf14528,	888371	recA	; O34519; P0A5	0	High	1	
O06817	Probable biotin sulfoxide reductase BisC (BD	MQVYTSATHWGVFTARVHGGDI		0.185	3	1	3	1	766	83.3	6.9		0		1	other metabolic processes	other cell component	her molecular functi	, Pf01568,	886620	bisC	; mtu:Rv1442;	0	High	1	
O53930	Possible integral membrane protein OS=Myc	MCAHEYAEQRSAVSGIEGLLTW		0.147	2	1	2	1	597	63.5	6.89		0		1		other membranes		Pf14362	885829		; L0T7N5; mtu:I	0	High	1	
P9WFJ5	UPF0603 protein Rv2345 OS=Mycobacterium	MRLVRLLGMVLTILAAGLLLGPP		0.174	2	1	3	1	660	70	8.38		1.66		1				Pf04536	888960		345; P95241; F	0	High	1	
I6YCF3	Probable helicase HelZ OS=Mycobacterium t	MLVLHGFWSNSGGMRLWAEDS	0	0	1	1	14	1	1013	111.5	5.9	500.88	6.96	1	1	cell organization and biogenesis		ding activity;other m	, Pf00271,	888635	helZ	3; L0TBI9; mtu:I	0	High	1	
O86320	Exopolyphosphatase OS=Mycobacterium tub	MRIGNCSGFYGDRLSAMREMLT		0.111	7	1	3	1	560	59.1	5.53		2.35		1				Pf07287	885860		; mtu:Rv0976c;	0	High	1	
P9WQ79	4-aminobutyrate aminotransferase OS=Myco	MASLQQSRRLVTEIPGPASQALT		0.19	3	1	1	1	449	46.8	5.59		1.72		1	other metabolic processes	cytosol	her molecular functi	Pf00202	6591; 88	gabT	589; P63504; F	0	High	1	
P9WGI1	RNA polymerase sigma factor SigA OS=Myco	MAATKASTATDEPVKRTATKSPA		0.111	2	1	1	1	528	57.8	4.86		0		1	cription;other metabolic processe	other cell component	ding activity;other m	4539, Pf04	6691; 88	sigA	3495; O08513;	0	High	1	
P9WF25	Antitoxin RelJ OS=Mycobacterium tuberculos	MSISASEARQRLFPLIEQVNTDH	0	0	90	79	3988	79	91	10.2	5.03	170913.4	2063.84	61	74	other biological processes		leic acid binding act	Pf02604	7356; 88	relJ	3357; O50386; I	0	High	1	Phospho [T21(100); S55(100); S74(99.6)]
P9WP93	Cobalamin biosynthesis protein CobD OS=M	MFASTWQTRAVGVLIGCLLDVVF		0.231	8	1	1	1	313	33	11		2.03		1	ther metabolic processes;transpo		activity;other molecu	Pf03186	887513	cobD	:Rv2236c; P9W	0	High	1	
P9WP21	2,3,4,5-tetrahydropyridine-2,6-dicarboxylate N	MSTVTGAAGIGLATLAADGSVLD		0.1	11	1	2	1	317	32.6	5.97		2.24		1	other metabolic processes	other cell component	her molecular functi	4602, Pf14	886088	dapD	01c; O05302; F	0	High	1	Phospho [S203(100); S212(90)]
O53749	Conserved protein OS=Mycobacterium tuberc	MTGQNGQVARISPGKFRQLGPV	0.414	0.192	10	1	7	1	190	21.3	9.72	104.05	0	1	1	other biological processes		her molecular functi	Pf02627	886296		mtu:Rv0464c;	0	High	1	Phospho [S126(100); T136(100)]
Q50732	Long conserved protein OS=Mycobacterium t	MPLRPTQVSGTGRTRCAGRSGV	0.407		1	1	1	1	1140	124.5	5.85	125.2		1					, Pf08379,	887737		; mtu:Rv2566;	0	High	1	
I6XF60	Conserved alanine and arginine rich protein C	MTADEPRSDDSSGSAPQPAATF	0.429		2	1	1	1	450	49.8	6.6	109.4		1					Pf03993	6718; 88		XF60; mtu:Rv27	0	High	1	
P9WHL7	Phosphoribosylformylglycinamidine synthase	MSPLARTPRKTSVLDTVEHAATT		0.104	2	1	1	1	766	80.8	5		0		1	other metabolic processes	other cell component	her molecular functi	, Pf02769,	885358	purL	O06631; P0A5	0	High	1	Phospho [S648(100); S649(100); S651(100)]
P9WN45	1,4-alpha-glucan branching enzyme GlgB OS	MSRSEKLTGEHLAPEPAEMARL		0.2	3	1	111	1	731	81.7	5.73		0		1	tion and biogenesis;other metabo	cytosol;other cell componen	her molecular functi	, Pf02806,	886893	glgB	:Rv1326c; P9W	0	High	1	
P9WIQ1	UDP-galactopyranose mutase OS=Mycobact	MQPMTARFDLFVVGSGFFGLTIA	0.182	0.267	3	1	3	1	399	45.8	5.81	219.67	1.84	1	1	enesis;other metabolic processes	cytosol	her molecular functi	3275, Pf13	886142	glf	809c; O06934;	0	High	1	
P71635	CRISPR system endoribonuclease Csm6 OS	MILFSPIGTADPITALGDGPMLHIV	0.32	0.235	2	1	4	1	415	46.2	6.48	153.19	1.81	1	1	stress response		her molecular functi	Pf09659	888510	csm6	; mtu:Rv2818c;	0	High	1	
O07254	HTH tetR-type domain-containing protein OS	MARSIPADRFSAIVAASARVFIAH	0.154	0.083	4	1	2	1	246	25.9	6.05	216.75	2.19	1	1	ism OR transcription;other biolog		ding activity;other m	Pf00440	4297; 88		mtu:Rv0330c;	0	High	1	
P95125	Carboxylic ester hydrolase LipN OS=Mycoba	MTKSLPGVADLRLGANHPRMWT	0.211		3	1	4	1	376	40.1	6.4	179.21		1			other cell component	her molecular functi	Pf07859	6959; 88	lipN	254; mtu:Rv29	0	High	1	
A0A089QRB	Mycolipanoate synthase OS=Mycobacterium	MRTATATSVAVIGMACRLPGGID		0.128	1	1	1	1	2085	220.3	5.57		0		1	other metabolic processes	ane;other membranes;other	her molecular functi	, Pf02801,	6055; 886	msl3	K5; L0T647; mtu	0	High	1	
O07434	Copper-sensing transcriptional repressor RicR	MTAAHGYTQQKDNYAKRLRRVE		0	10	1	5	1	96	10.4	8.53		3.42		1	ism OR transcription;other biolog	other cell component	ding activity;other m	Pf02583	4161; 88	ricR	0190; mtv:RVB	0	High	1	
O06319	Phospholipase Culp4 OS=Mycobacterium tub	MIPRPQPHSGRWRAGAARRLTS	0.5		8	1	1	1	226	23.1	9.16	44.92		1		other metabolic processes	acellular;plasma membrane	her molecular functi	Pf01083	7442; 88	cut4	G67; mtu:Rv34	0	High	1	
O53238	Probable transcriptional regulatory protein OS	MRQHSGIGVLDKAVGVLHAVAE		0.132	9	1	1	1	233	24.3	8.95		0		1	ism OR transcription;other biolog		ding activity;other m	1614, Pf09	6978; 88		KFV1; mtu:Rv29	0	High	1	Phospho [S175(100)]
P9WKG5	2-C-methyl-D-erythritol 2,4-cyclodiphosphate	MNQLPRVGLGTDVHPIEPGRPC		0.135	6	1	1	1	159	16.4	6.67		0		1	other metabolic processes		her molecular functi	Pf02542	7569; 88	ispF	581c; P65183;	0	High	1	
O50455	Magnesium transport protein CorA OS=Myco	MFPGFDALPEVLRPVARPQPPN	0.25	0.167	2	1	4	1	366	41.5	5.76	158.81	1.64	1	1	organization and biogenesis;tran		activity;other molecu	Pf01544	887106	corA	7P6; mtu:Rv123	0	High	1	
P9WFP9	UPF0047 protein Rv2556c OS=Mycobacteriu	MDTDVLDVDTARRRIVDLTDAVR		0.139	9	1	1	1	133	14.3	5.26		0		1				Pf01894	887451		556c; P67121;	0	High	1	
P9WQB1	Alanine dehydrogenase OS=Mycobacterium	MRVGIPTETKNNEFRVAITPAGV		0.143	6	1	1	1	371	38.7	6.23		0		1	metabolic processes;stress resp	non-structural extracellular	her molecular functi	1262, Pf05	6769; 88	ald	780; O33322; F	0	High	1	Phospho [T60(100)]
P9WFV7	Glycine--tRNA ligase OS=Mycobacterium tub	MHHPVAPVIDTVVNLAKRRGFVY		0.172	4	1	3	1	463	52.9	6.3		0		1	metabolism OR transcription;ot	extracellular;cytosol;other c	her molecular functi	0587, Pf03	6344; 88	glyQS	357c; O65932;	0	High	1	
I6Y479	Probable conserved transmembrane protein	MSGIASAALILSLALVVLPGSPRC		0.102	5	1	1	1	266	27.6	11.6		0		1		ma membrane;other membr		Pf00482	885618		479; mtu:Rv36	0	High	1	

Table S4. LCMS/MS of RelK T77A mutant

Master	Accession	Description	Sequence	Coverage [%]	# Peptides	# PSMs	# Unique Peptides	# AAs	MW [kDa]	calc. pI	Score MS Amanda 2.0: MS Amanda 2.0	Score Sequest HT: Sequest HT	# Peptides (by Search Engine): MS Amanda 2.0	# Peptides (by Search Engine): Sequest HT	Biological Process	Cellular Component	Molecular Function	Pfam IDs	Entrez Gene ID	Gene Symbol	Gene ID	Reactome Pathways	# Protein Pathway Groups	Found in Sample: [S13] F13: Sample	# Protein Groups	Modifications
Master Protein	P9WGY5	DNA-directed RNA polymerase subı	MSISQSDASLAAVPAVDQFDPSS(	40	1	1	1	110	11.8	4.46		3.27		1	ranscription;oth	ol;other cell comp	ıg activity;other	Pf01192	5368; 88	rpoZ	L0T993; mtu:Rv1390; P66721; P71660; P9WGY5	ıicrobial action and antimicrobial resistance	1	High	1	Phospho [S48(99.9); S49(99.9)]
Master Protein	P9WI65	Serine/threonine-protein kinase Pkn	MTDVDPHATRRDLVPNIPAELLEA	4	4	8	4	1110	119.3	5.81	792.26	4	4	2	tabolic process	ılar;plasma meml	ıity;other molecı	069, Pf1	888659	pknK	L0TEA2; mtu:Rv3080c; P95078; P9WI65		0	High	1	
Master Protein	P9WH63	30S ribosomal protein S12 OS=Myc	MPTIQQLVRKGRRDKISKVKTAAL	6	1	2	1	124	13.8	11.37	192.6	1.75	1	1	tabolic process	nslational appara	ıg activity;other	Pf00164	4644; 88	rpsL	tu:Rv0682; P41196; P9WH63; Q933X1; Q93MR7; Q93MI		0	High	1	
Master Protein	I6Y498	Probable anion transporter ATPase	MSVTPKTLDMGAILADTSNRVVVC	2	1	2	1	386	41.4	6.09		2.15		1			ır molecular fun	Pf02374	885317		I6Y498; mtu:Rv3680		0	High	1	
Master Protein	P9WIF7	Uncharacterized PE-PGRS family pı	MSFVIAAPETLVRAASDLANIGSTL	10	1	1	1	603	49.5	4.37		3.48		1				Pf00934	886899	'E_PGRS2	L0T9A9; mtu:Rv1325c; P9WIF7; Q10637		0	High	1	
Master Protein	P71552	Possible magnesium chelatase OS=	MSPSNLPRTVGELRAAGHRERGV	1	1	1	1	459	49.6	5.38	224.59		1		biological proc		ıity;other molecı	Pf00158	885405		I6XA76; L0T5E7; mtu:Rv0958; P71552		0	High	1	
Master Protein	P9WKJ3	Putative acetolactate synthase smal	MSPKTHTLSVLVEDKPGVLARVA/	13	1	1	1	168	18.2	8.16		0		1	metabolic proc	cytosol	ır activity;other ı	842, Pf1	6992; 88	ilvH	L0TE14; mtu:Rv3002c; O53249; P65161; P9WKJ3		0	High	1	Phospho [T166(95.5)]
Master Protein	I6YCF3	Probable helicase HelZ OS=Mycoba	MLVLHGFWSNSGGMRLWAEDSD	1	1	4	1	1013	111.5	5.9	179.6	5.49	1	1	nization and bi		ıg activity;other	Pf00271	888635	helZ	I6YCF3; L0TBI9; mtu:Rv2101		0	High	1	
**Master Protein**	**P9WF09**	**Toxin RelKT77A OS=Mycobacteri**	**MRSVNFDPDAWEDFLFWLAADRI**	**51**	**8**	**30**	**8**	**85**	**10.1**	**6.55**	**1493.75**	**37.64**	**4**	**7**	**ıer metabolic**		**cid binding act**	**Pf06769**	**7357; 88**	**relKT77A**	**L0TCJ4; mtu:Rv3358; O50387; P64528; P9WF09**		**0**	High	**1**	
Master Protein	P9WJZ1	Probable S-adenosylmethionine-dep	MCAFVPHVPRHSRGDNPPSASTA	4	1	1	1	274	30	5.36	184.85		1		metabolic proc		ır molecular fun	Pf08241	888604		L0TE97; mtu:Rv3030; O53277; P9WJZ1; Q7D695		0	High	1	Phospho [S219(100)]
Master Protein	Q79FI8	PPE family protein PPE35 OS=Myc	MHYSVLPPEINSALIFAGAGSGPM	4	1	1	1	987	97.9	4.28		3.27		1	biological proc			823, Pf0	885506	PPE35	F2GH77; I6YBY4; L0TAT2; mtu:Rv1918c; Q79FI8		0	High	1	

Table S5. Strains and Plasmids used in the study.

Strain or Plasmid	Description	Source or Reference
**Strains**		
E. coli DH5α	Δ*(argF-lac)169*, φ80d*lacZ58*(M15), Δ*phoA8*, *glnX44*(AS), *deoR481*, *rfbC1*, *gyrA96* (NalR), *recA1*, *endA1*, *thiE1* and *hsdR17*	Lab Collection
E. coli BL21 (DE3)	F^{-} *ompT gal dcm lon* $hsdS_B(rB^{-}mB^{-})$ λ(DE3 [*lacI lacUV5-T7p07 ind1 sam7 nin5*]) $[malB^{+}]_{K\text{-}12}(\lambda^{S})$	Lab Collection
M. smegmatis mc^2155	*ept-1*	Lab Collection
**Plasmids**		
pUAB100	*oriM oriE hsp60*-GCN4-Gly10-mDHFR [F1,2], Hyg^R	(2)
pUAB200	*attP int hsp60*-GCN4-Gly10-mDHFR [F3], Kan^R	(2)
pUAB300	*oriM oriE hsp60*-mDHFR[F1,2]-Gly10 -MCS, Hyg^R	(2)
pUAB400	*attP int hsp60*-mDHFR [F3]-Gly10-MCS, Kan^R	(2)
pUAB100::*relB*	*relB* cloned in BamHI-ClaI sites of pUAB100, creating $RelB_{[F1,2]\text{-}C}$	(3)
pUAB200::*relE*	*relE* cloned in MfeI-ClaI sites of pUAB200, creating $RelE_{[F3]\text{-}C}$	(3)
pUAB300::*relB*	*relB* cloned BamHI-ClaI sites of pUAB300, creating $RelB_{[F1,2]\text{-}N}$	(3)
pUAB400::*relE*	*relE* cloned in MfeI-ClaI sites of pUAB400, creating $RelE_{[F3]\text{-}N}$	(3)
pUAB300::*pknK*	*pknK* cloned in HindIII site of pUAB300, creating $PknK_{[F1,2]\text{-}N}$	(4)
pUAB400::*pknK*	*pknK* cloned in HindIII site of pUAB400, creating $PknK_{[F3]\text{-}N}$	(4)
pLTA(*relJ*)20	*relJ* cloned in BsaI sites of pLTAExp20, Tet-inducible, Amp^R	(5)
pCAK(*relK*)10	*relK* cloned in BsaI sites of pCAKExp10, Ara-inducible, Kan^R	(5)
pYA1556	PknK-pETSUMO expression vector, (His-6) tag, Kan^R	(6)
pYA1659	PknK-K55M-pETSUMO expression vector, (His-6) tag, Kan^R	(7)
pJFR19	Mycobacterial integrating vector having 3-kb amidase promoter, Hyg^R	(8)
pVLExp4231	IPTG inducible Expression vector, N-Terminal (His-10) tag, Amp^R	(9)
pVLExp::*relB*	*relB* cloned in NheI and Bsu36I sites of pVLExp4231	Lab collection
pVLExp::*relE*	*relE* cloned in NheI and Bsu36I sites of pVLExp4231	Lab collection
pVLExp::*relF*	*relF* cloned in NheI and Bsu36I sites of pVLExp4231	Lab collection
pVLExp::*relG*	*relG* cloned in NheI and Bsu36I sites of pVLExp4231	Lab collection
pVLExp::*relJ*	*relJ* cloned in NheI and Bsu36I sites of pVLExp4231	Lab collection
pVLExp::*relK*	*relK* cloned in NheI and Bsu36I sites of pVLExp4231	Lab collection
pUAB300::*relF*	*relF* cloned in BamHI-ClaI sites of pUAB300, creating $RelF_{[F1,2]\text{-}N}$	This study
pUAB400::*relG*	*relG* cloned in MfeI-ClaI sites of pUAB400, creating $RelG_{[F3]\text{-}N}$	This study
pUAB300::*relJ*	*relJ* cloned in BamHI-ClaI sites of pUAB300, creating $RelJ_{[F1,2]\text{-}N}$	This study
pUAB400::*relK*	*relK* cloned in MfeI-ClaI sites of pUAB400, creating $RelK_{[F3]\text{-}N}$	This study
pVLExp::*relK*T77A	Thr77 of *relK* replaced with Alanine in pVLExp::*relK*	This study
pCAK(*relK*T77A)10	Thr77 of *relK* replaced with Alanine in pCAK(*relK*)10	This study
pCAK(*relK*T77E)10	Thr77 of *relK* replaced with Glutamate in pCAK(*relK*)10	This study
pJFR19::$relJK_{WT}$	*relJK* coding region cloned in NdeI-XbaI sites of pJFR19	This study
pJFR19::$relJK_{Mut}$T77A	Thr77 of *relK* replaced with Alanine in pJFR19::*relJK*	This study
pJFR19::$relJK_{Mut}$T77E	Thr77 of *relK* replaced with Glutamate in pJFR19::*relJK*	This study

Supporting Experimental Procedures.

Protein Expression and Purification.

E. coli BL21 (DE3) cells were transformed with the plasmids listed in Table S5. Primary cultures were set up from glycerol stocks and grown overnight at 37℃, 180 rpm in 5 mL LB Broth supplemented with Ampicillin or Kanamycin as per requirement. 1 % of primary culture inoculum was used to inoculate 400 mL of 2xYT Broth and grown until OD_{600nm} reached 0.4-0.6. Then the culture was kept in 4°C for 30 mins, followed by IPTG induction at a final concentration of 1 mM. The cultures were then incubated for 16-18 hours at 16°C, 180 rpm. The cells were harvested by centrifuging at 5000 rpm for 10 mins at 4℃.

As described previously (4) , for native proteins, the pellets were resuspended in 10 mL Lysis Buffer (50 mM Tris-HCl pH 8.0, 500 mM NaCl, 10 mM Imidazole, and Protease Inhibitor cocktail). Resuspended cells were then sonicated for 30 mins at 35 % amplitude in cycles of 5 secs ON and 5 secs OFF. The cell suspension was centrifuged at 13000 rpm for 30 mins at 4°C. The supernatant was loaded onto the Ni^{+2}-NTA column pre-equilibrated with native Lysis Buffer and incubated for 16-18 hours at 4°C with constant agitation for the protein to bind to the beads. The column was then washed with wash buffers (50 mM Tris-HCl pH 8.0, 300 mM NaCl, 20 mM – 40 mM Imidazole) and protein was eluted in fractions of 1 mL using Elution Buffer (50 mM Tris-HCl pH 8.0, 300 mM NaCl and 500 mM Imidazole). The fractions were pooled and buffer exchange was done using protein storage buffer (50 mM Tris-HCl pH 8.0, 250 mM NaCl, 50 % glycerol). The protein concentration after buffer exchange was estimated by Bradford assay using BSA as standard and purity of the proteins were checked on SDS-PAGE. The purified proteins were stored at -20°C.

As described previously (10) , for purification of proteins under denaturing conditions, the pellet was resuspended in 10 mL of Denaturing / Solubilization Buffer (20 mM Tris-HCl pH 8.0, 500 mM NaCl, 10 % Glycerol, 8M Urea). The pellet was incubated at 37°C with shaking for 2 hours. The solubilized pellet was centrifuged at 12000g, 4°C for 15 minutes. The supernatant was loaded onto the Ni^{+2}-NTA column pre-equilibrated with Denaturing / Solubilization Buffer and incubated for 16-18 hours at 4°C with constant agitation for the protein to bind to the beads. Then, column was washed with decreasing concentration of urea (6M to 1M) in Refolding Buffer (20 mM Tris-HCl pH 8.0, 500 mM NaCl, 10 % Glycerol, 5 mM Reduced Glutathione) followed by wash with Refolding Buffer without urea. 1 mL fractions of the proteins were eluted using 10 mL Refolding Buffer with 250 mM Imidazole. The fractions were pooled and buffer exchange was done using protein storage buffer (50 mM Tris-HCl pH 8.0, 250 mM NaCl, 50 % glycerol). The protein concentration after buffer exchange was estimated by Bradford assay using BSA as standard and purity of the proteins were checked on SDS-PAGE. The purified proteins were stored at -20°C.

Identification of Phosphosites by Mass spectrometry.

Mass spectrometry was performed by Valerian Chem Pvt. Ltd., New Delhi as follows; **Sample Preparation:** Gel band were cut into small pieces and reduced with 5 mM TCEP and further alkylated with 50 mM iodoacetamide and then digested with Trypsin (1:50, Trypsin/lysate ratio) for 16 h at 37 °C. Digests were cleaned using a C18 silica cartridge to remove the salt and dried using a speed vac. The dried pellet was resuspended in buffer A (2 % acetonitrile, 0.1 % formic acid). **Mass Spectrometric Analysis of Peptide Mixtures:** Experiments were performed on an Easy-nlc-1000 system coupled with an Orbitrap Exploris mass spectrometer. 1 µg of peptide sample were loaded on C18 column 15 cm, 3.0µm Acclaim PepMap (Thermo Fisher Scientific)

and separated with a 0–40 % gradient of buffer B (80 % acetonitrile, 0.1 % formic acid) at a flow rate of 300 nL/min) and injected for MS analysis. LC gradients were run for 110 minutes. MS1 spectra were acquired in the Orbitrap (Max IT = 60 ms, AGQ target = 300 %; RF Lens = 70 %; R=60K, mass range = 375−1500; Profile data). Dynamic exclusion was employed for 30 s excluding all charge states for a given precursor. MS2 spectra were collected for top 20 peptides. MS2 (Max IT= 60 ms, R= 15K, AGC target 100 %). **Data Processing:** All samples were processed, and RAW files generated were analyzed with Proteome Discoverer (v2.5) against the Uniprot *M. tb* H37Rv database. For dual Sequest and Amanda search, the precursor and fragment mass tolerances were set at 10 ppm and 0.02 Da, respectively. The protease used to generate peptides, i.e., enzyme specificity was set for trypsin/P (cleavage at the C terminus of “K/R: unless followed by “P”). Carbamidomethyl on cysteine as fixed modification and oxidation of methionine and N-terminal acetylation were considered as variable modifications for database search. Both peptide spectrum match and protein false discovery rate were set to 0.01 FDR.

All-atomistic Molecular Dynamics Simulations of the RelJK TA module. Charmm GUI (11, 12) Input Generator was used for the obtaining the main parameter files (i.e. ITP and TOP files), which are required to initiate the simulation process in the Gromacs environment for RelK and RelK~P. The molecular dynamics simulations for the RelK, phosphorylated RelK (RelK~P), RelJ and the RelJK complexes were conducted utilizing the CHARMM36 (13) force fields. Gromacs software was employed to execute these molecular dynamics simulations. The individual chains and complexes were solvated on the TIP3P model (14) in the centre of an octahedron or dodecahedron cell at a distance of 1.0 nm from the edge of the box.

The topology files for the chains and the complexes were generated using the Charmm GUI and Gromacs pdb2gmx command. An appropriate balancing counter ion (K^+ or Cl^-) was added to neutralize the system. Particle mesh Ewald was used to treat long-range electrostatic interactions (15). The pressure and temperature were controlled with the Parrinello-Rahman coupling algorithm, Nose Hoover (16, 17) and V-rescale (modified Berendsen) thermostat (18). To avoid the steric clashes, the solvated and neutralized system was subject to relaxation through Steepest Descent (SD) minimization algorithm with a maximum of 5000 steps for RelK chain simulations and 50000 steps for RelJ chain simulations. Then, the system was equilibrated with 125 ps of isothermal-isochronic (NVT) at 303.15 K for RelK chain simulations and RelJ chain was equilibrated for 100 ps at 300.00 K followed by equilibration using isothermal-isochronic (NPT) ensemble at a pressure of 1 atm for 100 ps. Linear Constraint Solver (LINCS) algorithm (19) was used to converge the bond angles and length in the system. Finally, the production MD run of 250 ns was performed for protein chains (Toxin or Antitoxin) and 200 ns for the TA complexes. The structures were visualized and rendered using UCSF Chimera (20).

	# of protein atoms	# of ion	# of water atoms	# of atoms in the Total system size	Temperature (K)	Time (ns)
RelJ	1345	3	58,644	59,992	300.00	250
RelK	1402	23	12,579	14,004	303.15	250
RelK~P	1406	24	12,570	14,000	303.15	250
RelJK	2747	4	27,918	30,669	300.00	200
RelJK~P	2751	5	71,574	74,330	300.00	200

References.

1. Frando, A., Boradia, V., Gritsenko, M., Beltejar, C., Day, L., Sherman, D. R., Ma, S., Jacobs, J. M., and Grundner, C. (2023) The Mycobacterium tuberculosis protein O-phosphorylation landscape. *Nature Microbiology*. **8**, 548–561
2. Singh, A., Mai, D., Kumar, A., and Steyn, A. J. C. (2006) Dissecting virulence pathways of *Mycobacterium tuberculosis* through protein–protein association. *Proc. Natl. Acad. Sci. U.S.A.* **103**, 11346–11351
3. Korch, S. B., Contreras, H., and Clark-Curtiss, J. E. (2009) Three *Mycobacterium tuberculosis* Rel Toxin-Antitoxin Modules Inhibit Mycobacterial Growth and Are Expressed in Infected Human Macrophages. *J Bacteriol*. **191**, 1618–1630
4. Malhotra, V., Okon, B. P., Satsangi, A. T., Das, S., Waturuocha, U. W., Vashist, A., Clark-Curtiss, J. E., and Saini, D. K. (2022) Mycobacterium tuberculosis PknK Substrate Profiling Reveals Essential Transcription Terminator Protein Rho and Two-Component Response Regulators PrrA and MtrA as Novel Targets for Phosphorylation. *Microbiol Spectr*. **10**, e01354-21
5. Gupta, A. (2009) Killing activity and rescue function of genome-wide toxin–antitoxin loci of *Mycobacterium tuberculosis*. *FEMS Microbiology Letters*. **290**, 45–53
6. Malhotra, V., Arteaga-Cortes, L. T., Clay, G., and Clark-Curtiss, J. E. (2010) Mycobacterium tuberculosis protein kinase K confers survival advantage during early infection in mice and regulates growth in culture and during persistent infection: implications for immune modulation. *Microbiology*. **156**, 2829
7. Malhotra, V., Okon, B. P., and Clark-Curtiss, J. E. (2012) Mycobacterium tuberculosis Protein Kinase K Enables Growth Adaptation through Translation Control. *J Bacteriol*. **194**, 4184–4196
8. Chauhan, A., Madiraju, M. V. V. S., Fol, M., Lofton, H., Maloney, E., Reynolds, R., and Rajagopalan, M. (2006) *Mycobacterium tuberculosis* Cells Growing in Macrophages Are Filamentous and Deficient in FtsZ Rings. *J Bacteriol*. **188**, 1856–1865
9. Chaudhary, V. K., Shrivastava, N., Verma, V., Das, S., Kaur, C., Grover, P., and Gupta, A. (2014) Rapid Restriction Enzyme-Free Cloning of PCR Products: A High-Throughput Method Applicable for Library Construction. *PLOS ONE*. **9**, e111538
10. Saini, D. K., Pant, N., Das, T. K., and Tyagi, J. S. (2002) Cloning, Overexpression, Purification, and Matrix-Assisted Refolding of DevS (Rv 3132c) Histidine Protein Kinase of Mycobacterium tuberculosis. *Protein Expression and Purification*. **25**, 203–208
11. Jo, S., Kim, T., Iyer, V. G., and Im, W. (2008) CHARMM-GUI: A web-based graphical user interface for CHARMM. *J Comput Chem*. **29**, 1859–1865
12. Brooks, B. R., Brooks, C. L., Mackerell, A. D., Nilsson, L., Petrella, R. J., Roux, B., Won, Y., Archontis, G., Bartels, C., Boresch, S., Caflisch, A., Caves, L., Cui, Q., Dinner, A. R., Feig, M., Fischer, S., Gao, J., Hodoscek, M., Im, W., Kuczera, K., Lazaridis, T., Ma, J., Ovchinnikov, V., Paci, E., Pastor, R. W., Post, C. B., Pu, J. Z., Schaefer, M., Tidor, B., Venable, R. M., Woodcock, H. L., Wu, X., Yang, W., York, D. M., and Karplus, M. (2009) CHARMM: The biomolecular simulation program. *J Comput Chem*. **30**, 1545–1614
13. Huang, J., and MacKerell, A. D. (2013) CHARMM36 all-atom additive protein force field: Validation based on comparison to NMR data. *J. Comput. Chem.* **34**, 2135–2145

14. Jorgensen, W. L., Chandrasekhar, J., Madura, J. D., Impey, R. W., and Klein, M. L. (1983) Comparison of simple potential functions for simulating liquid water. *The Journal of Chemical Physics*. **79**, 926–935
15. Darden, T., York, D., and Pedersen, L. (1993) Particle mesh Ewald: An $N \cdot \log(N)$ method for Ewald sums in large systems. *The Journal of Chemical Physics*. **98**, 10089–10092
16. Hoover, W. G. (1985) Canonical dynamics: Equilibrium phase-space distributions. *Phys. Rev. A*. **31**, 1695–1697
17. Nosé, S. (1984) A unified formulation of the constant temperature molecular dynamics methods. *The Journal of Chemical Physics*. **81**, 511–519
18. Bussi, G., Donadio, D., and Parrinello, M. (2007) Canonical sampling through velocity rescaling. *The Journal of Chemical Physics*. **126**, 014101
19. Hess, B., Bekker, H., Berendsen, H. J. C., and Fraaije, J. G. E. M. (1997) LINCS: A linear constraint solver for molecular simulations. *J. Comput. Chem.* **18**, 1463–1472
20. Pettersen, E. F., Goddard, T. D., Huang, C. C., Couch, G. S., Greenblatt, D. M., Meng, E. C., and Ferrin, T. E. (2004) UCSF Chimera—A visualization system for exploratory research and analysis. *J Comput Chem*. **25**, 1605–1612
